# Reactive Heterobimetallic Complex Combining Divalent Ytterbium and Dimethyl Nickel Fragments

**DOI:** 10.3390/inorganics7050058

**Published:** 2019-04-26

**Authors:** Ding Wang, Jules Moutet, Maxime Tricoire, Marie Cordier, Grégory Nocton

**Affiliations:** LCM, CNRS, Ecole Polytechnique, IP Paris, Route de Saclay, 91128 Palaiseau, France

**Keywords:** divalent lanthanides, redox non-innocent ligand, magnetism, CO insertion

## Abstract

This article presented the synthesis and characterization of original heterobimetallic species combining a divalent lanthanide fragment and a divalent nickel center bridged by the bipyrimidine ligand, a redox-active ligand. X-ray crystal structures were obtained for the Ni monomer (bipym)NiMe_2_, **1**, as well as the heterobimetallic dimer compounds, Cp*_2_Yb(bipym)NiMe_2_, **2**, along with ^1^H solution NMR, solid-state magnetic data, and DFT calculations only for **1**. The reactivity with CO was investigated on both compounds and the stoichiometric acetone formation is discussed based on kinetic and mechanistic studies. The key role of the lanthanide fragment was demonstrated by the relatively slow CO migratory insertion step, which indicated the stability of the intermediate.

## 1 Introduction

Heterometallic complexes are important objects of study because both metallic fragments have a role to play in the chemical reaction and/or properties of interest [1–3]. In nature, the active sites of many enzymes are bimetallic, and the understanding of the role of each metallic part is crucial to the design of appropriate models [4–7]. In some cases, the role of one fragment is purely structural and only facilitates the reaction at the other metal center, while in most cases, both fragments have a role and either participate in the fate of the chemical reaction or complete it [8]. This particular case was extensively studied in terms of the tandem-reaction catalysts, where, for example, one metal is the source of one reaction, the other of a second one, and the substrate undergoes two chemical transformations in one pot [9–12]. Another elegant use of the bimetallic complexes is found in the chemical cooperation between both metal fragments, allowing a reactivity that would not occur efficiently with only one of these taken separately [13]. The recent interest in photochemically active bimetallic complexes is witness to these developments [14,15].

In our group, we designed bimetallic complexes with a slightly different approach. We combined a reductive divalent lanthanide fragment with a transition metal fragment, which possessed a ligand that could be eventually reduced or oxidized upon coordination. These studies have recently led us to develop a system with Pd and the bipyrimidine ligand (bipym) in which the palladium can be stabilized at the Pd^IV^ for several hours at room temperature [16]. This result was rendered possible using divalent ytterbium, which can reduce the palladium/bipym complex and have an impact on the overall electronic structure of the bimetallic assembly. Thus, the two metallic fragments cooperate by means of their electronic correlation. The use of divalent lanthanide is purposeful, since as they are strong single electron reductants [17–20], the divalent lanthanides adapt their electronic structures depending on the ligand that is used and form multiconfigurational electronic states [21–23], which allow the tuning of their properties with the redox-active ligand [24–29].

High oxidation states such as Pd^IV^ or Ni^IV^ are actively sought after because of their scarcity, but also because they allow important reactions [30–34]. Notably, the oxidative addition from Pd^II^ or Ni^II^ centers is an important step because it may avoid the difficult use of low-valence Pd and Ni species as catalysts, and their electrophilic character could also prevent the use of other electrophile metal fragments which have less-abundant resources, such as Rh, Ir, or Pt [35,36]. For example, methanol is transformed to acetic acid using carbon monoxide (CO) in a reaction that uses Ir (Cativa) [37,38] or Pt (Monsanto) [39] catalysts. In these reactions, the crucial step of CO insertion is performed after the oxidative addition at the high oxidation state of the metal center [40].

In this work, we developed a new complex that possesses a Ni metal center and bipym as the ligand and combined it with the Cp*_2_Yb divalent lanthanide fragment. The synthesis and characterization of the bimetallic complex is presented, as well as reactivity studies implying CO insertion.

## 2 Results and Discussions

### 2.1 Synthesis and X-ray Diffraction

**Figure 1:**
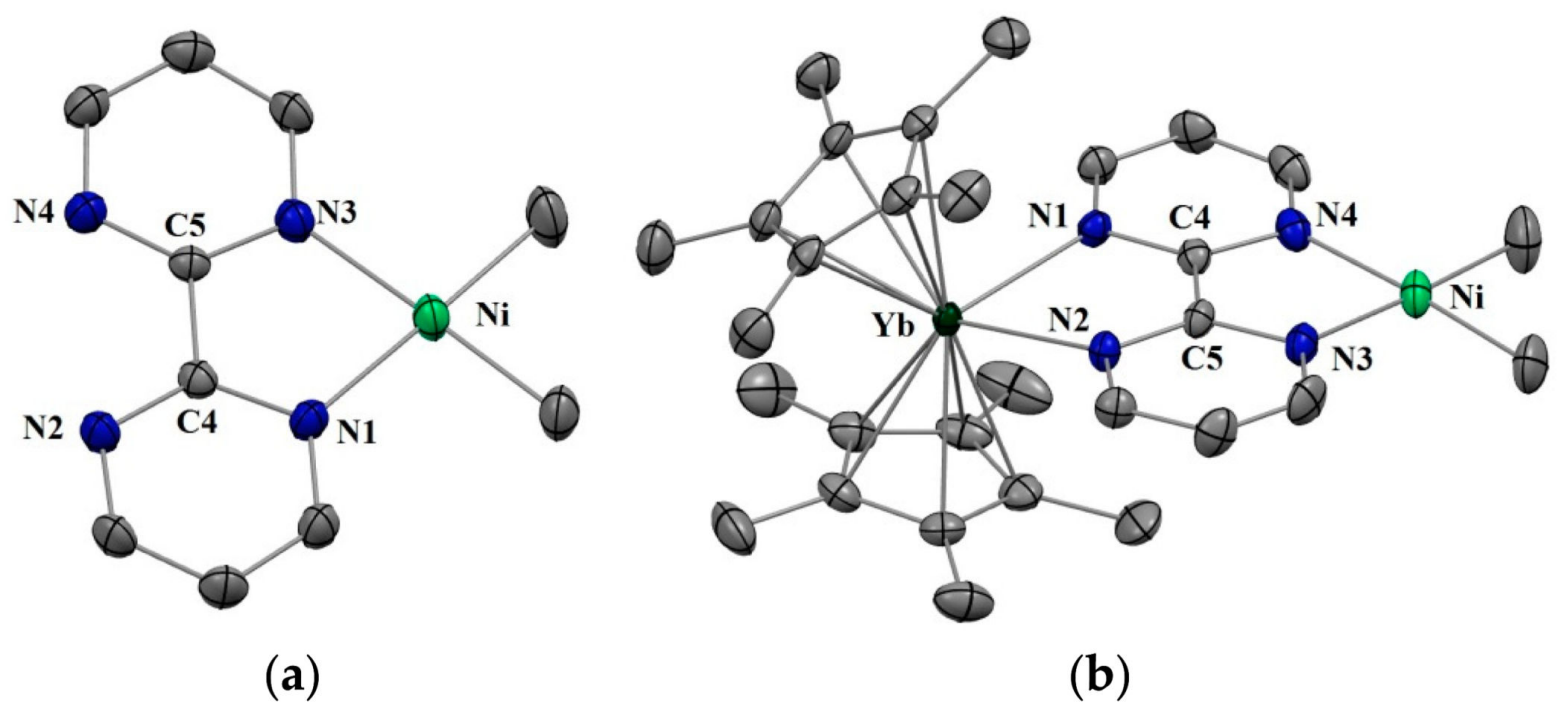
(**a**) Oak Ridge Thermal Ellipsoid Plot (ORTEP) of (bipym)NiMe_2_ (**1**) (only one of the molecules of the cell is shown) (**b**) ORTEP of Cp*_2_Yb(bipym)NiMe_2_ (**2**). Hydrogen atoms have been removed for clarity and thermal ellipsoids are at the 50% level.

**Scheme 1:**
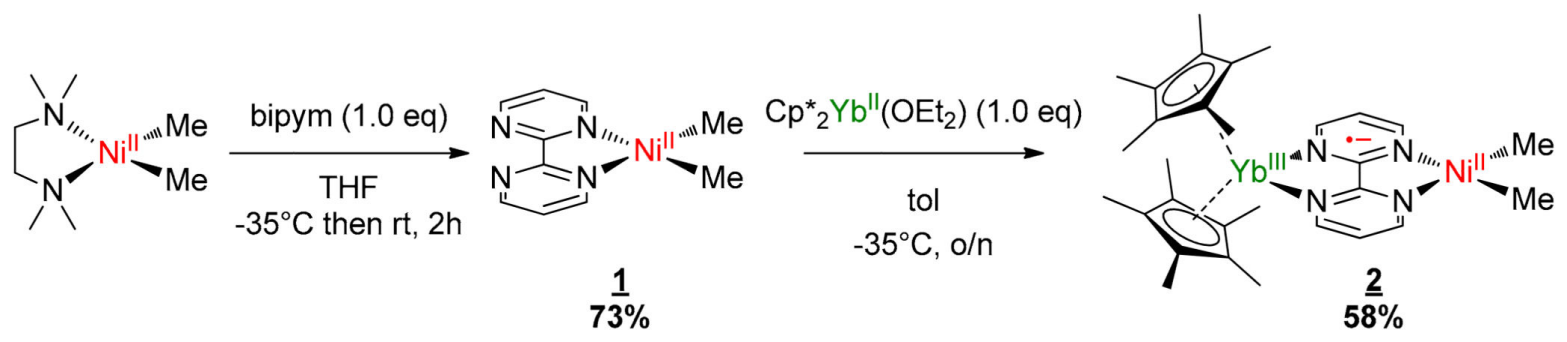
Synthesis of the bimetallic complex. bipym: bipyrimidine ligand.

Complexes **1** and **2** were synthesized according to similar published procedures for similar Pd complexes (Scheme 1) [16]. The (tmeda)NiMe_2_ [41] was suitable for the ligand exchange in THF with bipyrimidine (bipym) and the reaction yielded (bipym)NiMe_2_ (**1**) as dark X-ray-suitable crystals in good yield after the solution was cooled to −35 °C. Note that this synthesis method avoided the formation of the (bipym) (NiMe_2_)_2_ dimer as well as free bipym, which are both very problematic for the next step. An Oak Ridge Thermal Ellipsoid Plot (ORTEP) of **1** is shown in Figure 1, and the main metric parameters are available in Supplementary Materials. Complex **1** was dissolved in toluene and cooled down to −35 °C and the addition of a room temperature toluene solution of the Cp*_2_Yb(OEt_2_) complex led to a dark-brown solution, which yielded X-ray-suitable dark-brown crystals of the heterobimetallic dimer Cp*_2_Yb(bipym)NiMe_2_, **2**, when cooled down to −35 °C. An ORTEP is shown in Figure 1, and the main metric parameters are available in the Supplementary Materials. Comparing the solid-state structures of **1** and **2**, the first noteworthy feature is the C4–C5 distance, which was strongly reduced in **2** (1.403(4) Å) compared to that in **1** (1.482(5) Å). This is similar to the observation made with the Pd complex [16] and is due to an electron transfer from the ytterbium center that reduced the bipyrimidine ligand. Accordingly, the average Cp centroid–Yb distance was 2.31(1) Å, which is lower than that when the fragment is divalent and similar to that when it is trivalent [25]. The Ni–N (1.959(2) and 1.956(3) Å) and Ni–C (1.930(3) and 1.925(1) Å) average distances were similar in both **1** and **2**, respectively, which is indicative of a similar oxidation state in both complexes.

### 2.2 Solution NMR and Solid-State Magnetism

**Figure 2:**
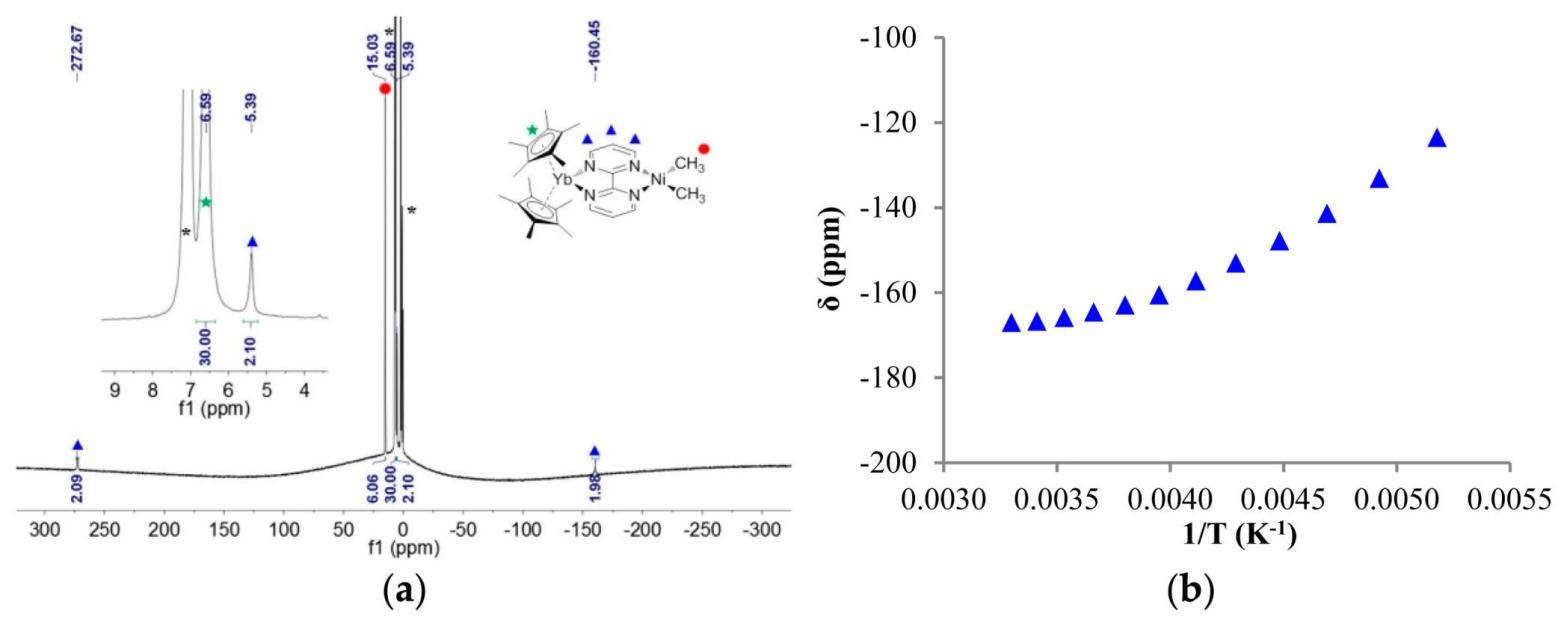
(**a**) ^1^H NMR of **2** at 253 K, solvent and grease impurities are indicated by an asterisk (*); (**b**) Variable temperature ^1^H NMR chemical shifts (δ) versus 1/T (K^−1^) of the proton at −160.5 ppm on the previous spectrum.

The ^1^H NMR of **1** showed only four signals: three for the bipym ligand, integrating each for two protons, and one for the methyl fragments, integrating for six protons, in good agreement with a C_2v_ symmetry in solution. In **1**, all signals were found in the typical diamagnetic range. The ^1^H NMR of **2** at 253 K in tol-*d*_8_ was very different: three signals integrated for two protons at 272.67, 5.39, and −160.45 ppm, one signal integrated for six protons at 15.03 ppm, and one for 30 protons at 6.59 ppm (Figure 2). The latter signal was easily attributed to the protons of the Cp* fragments, while the former were attributed to the bipym ligand and the methyl fragments, respectively. The spectrum is in agreement with a C_2v_ symmetry in solution, and the clear paramagnetism of the signals agrees with an electron transfer from the divalent ytterbium to the bipym ligand. In such a situation, the ytterbium metal center becomes trivalent and is f^13^ (one hole on the f-shell) while the bipym is a radical anion.

Considering the paramagnetism measured by NMR, multiple possibilities arise for the electronic ground state of **2**: a triplet state or a singlet state with a low-lying triplet state. The chemical shifts of each resonance were plotted versus 1/T (Figure S5), and an example is given in Figure 2. It is clear from these plots that the behavior did not strictly follow Curie’s law, since the plots are curved. Two reasons may explain this behavior: (i) the bipym ligand in **2** was in exchange with free bipym and the chemical shift depended on the equilibrium thermodynamics at a given temperature, or (ii) the magnetic behavior of **2** did not follow Curie’s law because of a magnetic exchange coupling between the two single electrons. To test these two propositions, the solid-state magnetism was measured.

**Figure 3:**
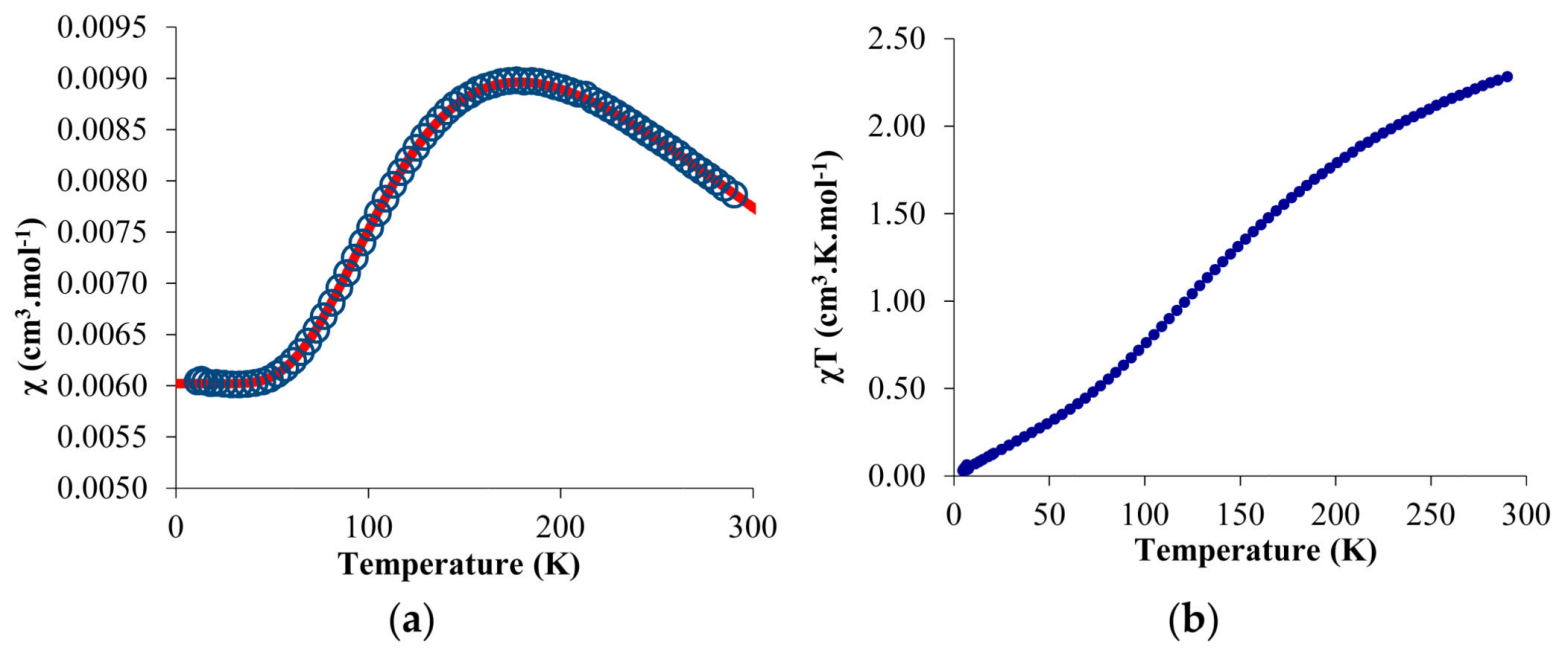
Solid-state temperature-dependent magnetic data at 0.5 T: (**a**) plot of *χ* versus T and of (**b**) *χ*^T^ versus T. The solid line represents the fit of the magnetic data (see text).

The temperature-dependent magnetic data were recorded and χ and χT were plotted versus T and are represented in Figure 3. The χT value decreased gradually with decreasing temperature until the value approached zero, with two inflection points around 65 and 170 K. The near-zero value at low temperature indicates a singlet ground state while the value of 2.3 emu·K·mol^−1^ is close to the theoretical value obtained by the sum of a ^2^F state (f^13^) and a ^2^S state (bipym·^−^). The χ value versus the temperature is more indicative of the magnetic behavior of **2**: a maximum was obtained at 170 K while the low-temperature data was low and independent of the temperature (temperature-independent paramagnetism (TIP) or Van Vleck paramagnetism) [42,43]. Such a behavior can be explained by an anti-ferromagnetic coupling between the single electron located on the ytterbium center and that on the bipym ligand. The TIP was indicative of a low-lying triplet state above the singlet ground state. The χT value at room temperature agrees with a substantial population of the triplet at this temperature and therefore a low-lying triplet excited state. Using a modified version of the Bleaney–Bowers equation (Equation (1)) [44], the data were fitted using an average g-value of 3.78, a TIP of 0.00602 emu·mol^−1^, and a *2J* value of −275.2 cm^−1^. The average g-value that was used was relatively close to the one measured for the similar trivalent ytterbium complexes, that is, 3.315 for Cp*_2_Yb(bipy)^+^ [45]. The red curve is the fit of the data with the Equation (1), and is very satisfactory. However, the value of both TIP and g-average are in very high agreement with a high anisotropy in this system. In order to account for the anisotropy, we used another model from Lukens et al. that is more adapted to highly anisotropic compounds such as the lanthanide complexes [46]. Assuming the c_1_^2^ is close to 1 in **2** (which means that the oxidation state is close to purely trivalent) and using the g-value of the similar Cp*_2_Yb(bipy)^+^ complex [45], the *2J* value was estimated to be -245.65 cm^−1^—a similar value to that found with the simple Bleaney–Bowers model (Equation (1)). It indicates that the exchange coupling was rather strong, compares well with the value found in the literature for the Cp*_2_Yb fragment and other N-aromatic heterocycles [46], and is typical for this type of species [47].

(1) $$\chi =\frac{\frac{2\text{N}\beta^{2}{\text{g}}_{\text{ave}}^{2}{\text{e}}^{\text{x}}}{\text{kT}}+\text{TIP}\left(1-{\text{e}}^{\text{x}}\right)}{\left(1+3{\text{e}}^{\text{x}}\right)},\;\text{x}=\frac{2\text{J}}{\text{kT}}$$

The magnetism as well as the ^1^H solution NMR spectroscopy agreed with a singlet ground state and a triplet state that is substantially populated. Strong spin density was located on the bipym ligand as indicated by the X-ray crystallography, and the exchange coupling was monitored by the solid-state magnetism. However, in order to probe the reactivity at the nickel center, it is important to quantify how the electronic density is organized on the transition metal. For this, we turned to theoretical computations on **1**.

### 2.3 Theoretical Computations on **1**

**Figure 4:**
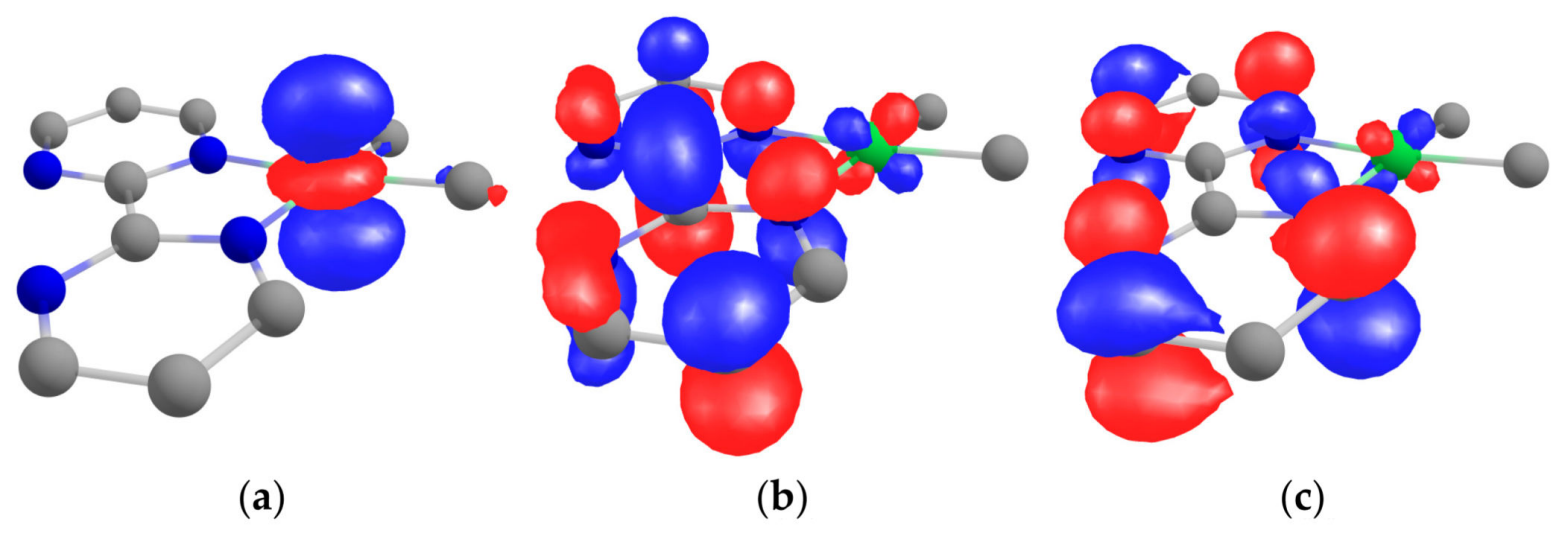
Kohn–Sham orbitals of **1** at the TPSSh level of theory calculated taking PBE optimized geometry: (**a**) HOMO, (**b**) LUMO, and (**c**) LUMO +1.

Theoretical computations were performed on **1** at the DFT level with three different functionals: PBE, PBE0, and TPSSh (see the Materials and Methods section for more information). The difference in geometry is given in the Supplementary Materials, and the differences were minor compared to the X-ray data (see Table S7). The HOMO was clearly the dz_2_ of the nickel center, while the LUMO and LUMO +1 were orbitals located in the bipym ligand with the b_1_ symmetry (in C_2v_) (Figure 4). The LUMO possessed much density on the C–C bond that linked the two pyrimidine rings, the LUMO +1 had a node at these positions. The density on the nickel center was only residual, and the estimated atomic orbital Mulliken contribution from the nickel to these two MOs were 8.3% for the LUMO and 4.6% for the LUMO +1.

According to the magnetism, the ground state of **2** was a singlet state while the triplet state was low in energy. We did not perform DFT computations on **2** because the electronic state was likely to be multiconfigurational considering the previous data published on similar compounds [21–25,27] and the multi-referential calculations are more difficult considering the number of atoms. However, the magnetism and the X-ray crystal structure were indicative of spin density localized on the C–C bond that linked the two pyrimidine rings, which was in agreement with an electron transfer from the Cp*_2_Yb fragment to the LUMO of **1**. Another interesting feature was the high-energy dz^2^ located on the Ni center that is ready for reactivity. However, the addition of MeI or MeOTf on **1** and **2** only led to the very fast formation of ethane, even at low temperature. If it is indicative of some reactivity, the lack of intermediates observed in the course of the reaction does not allow the drawing of any conclusions on the possible mechanism. Therefore, we turned to the reaction of **1** and **2** with CO.

### 2.4 Reactivity with Carbon Monoxide

**Scheme 2:**
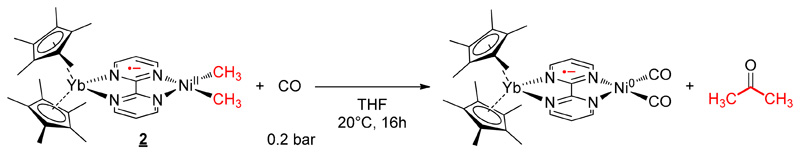
Reactivity of **2** with carbon monoxide.

The reactivity with CO and group 10 alkyl complexes has been well investigated over the years. We were interested in knowing whether the presence of the lanthanide fragment in **2** influenced the mechanism of such a reaction. Thus, the reactivity at a low concentration of CO (0.2 bar) was investigated in both complexes **1** and **2** in THF. The reaction occurred rapidly and was followed by ^1^H NMR spectroscopy. After a few minutes of reaction, in the case of complex **2**, new signals appeared at 259.9, 12.4, and −0.1 ppm, while the starting material concentration decreased slowly (see Figure S12). As the reaction time evolved, the intensity of these new signals increased then dropped until the end of the reaction. Similar to **2**, in the complex **1** system, new signals appeared at 9.2 and 2.2 ppm with time, and the rapid formation of acetone was clearly observed (see Figure S19). These new signals were attributed to the acyl intermediate after the CO migratory insertion in the Ni–C bond. The prior coordination of the CO on the nickel center through the loaded dz^2^ orbital was not observed. The formation of acetone (Scheme 2) was monitored with time. The apparition of this ketone indicates a reductive elimination from the acyl-methyl divalent nickel center to form Ni^0^ species. Considering the presence of CO in excess, the formation of bis-CO Ni^0^ species is likely. At the end of the reaction, the signals of the intermediates disappeared, and unfortunately, at this point, the reduced complex could not be isolated. Instead, the formation of a small amount of free bipym ligand as well as the Cp*_2_Yb(bipym)YbCp*_2_ [48] dimer was observed, indicating the disassociation of the reduced Ni^0^ complex.

**Figure 5:**
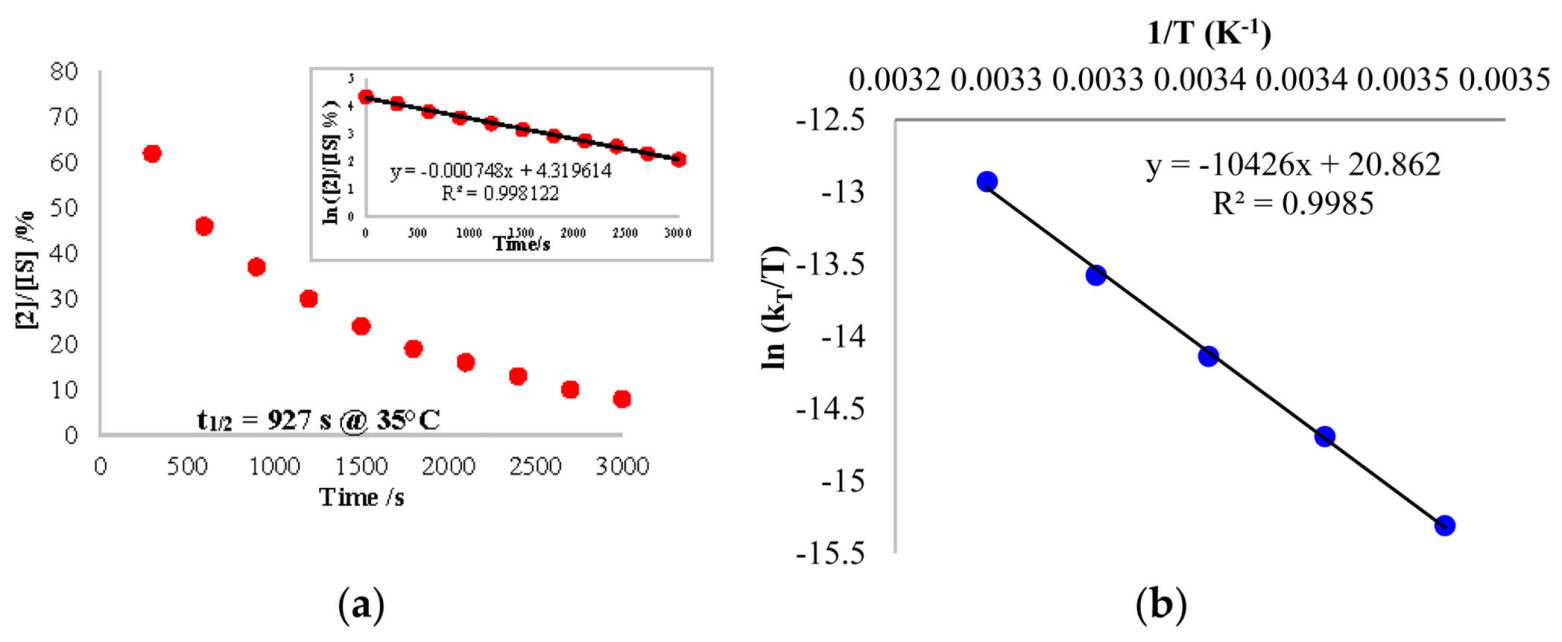
(**a**) Plot of the concentration of **2***/*IS (normalized) over the reaction time at 35 °C. The insert shows the kinetics order. (**b**) Eyring plot analysis for the reaction of **2** with carbon monoxide. The internal standard (IS) used was the toluene molecules crystallized in the cell.

**Scheme 3:**
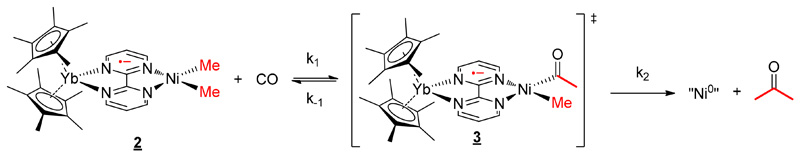
Mechanism for the reaction of **2** with CO.

The reaction kinetics was followed by analysis with ^1^H NMR spectroscopy, using the decrease of the signal of **2**. A mono-exponential variation decrease of the concentration of **2** was recorded at 35 °C (Figure 5a). The variation of the concentration of **2** did not modify the observed rate of the reaction at the same temperature at moderate concentrations of **2** (0.0073–0.029 mol/L). At higher concentrations, there was no longer a large excess of CO and the rate evolved. This information indicates that the rate overall order was pseudo first-order in **2** only when CO was in excess. The mechanism as well as the rate law can be written accordingly (Scheme 3 and Equation (2)):

(2) $$\text{r}=\frac{k_{1}k_{2}\left[2\right]\left[\text{CO}\right]}{k_{-1}+k_{2}}$$

Using the rate law, the same analysis over a temperature range from 15 to 35 °C allowed us to perform an Eyring analysis (Figure 5b). The activation parameters obtained were 20.7 kcal·mol^−1^ and –5.75 cal·mol^−1^·K^−1^. The moderate ∆H^≠^ is in agreement with a rather slow reaction at room temperature while the small negative ∆S^≠^, indicates that the reductive elimination (entropically favorable) was not the rate-determining step (RDS) or it was balanced with a step with similar rate that was not favorable in entropy. On the other hand, the migratory insertion had a more modest effect on the entropy and could correspond to the RDS in this case. Without the information on the C coordination, it is not possible to conclude at this stage.

**Scheme 4:**
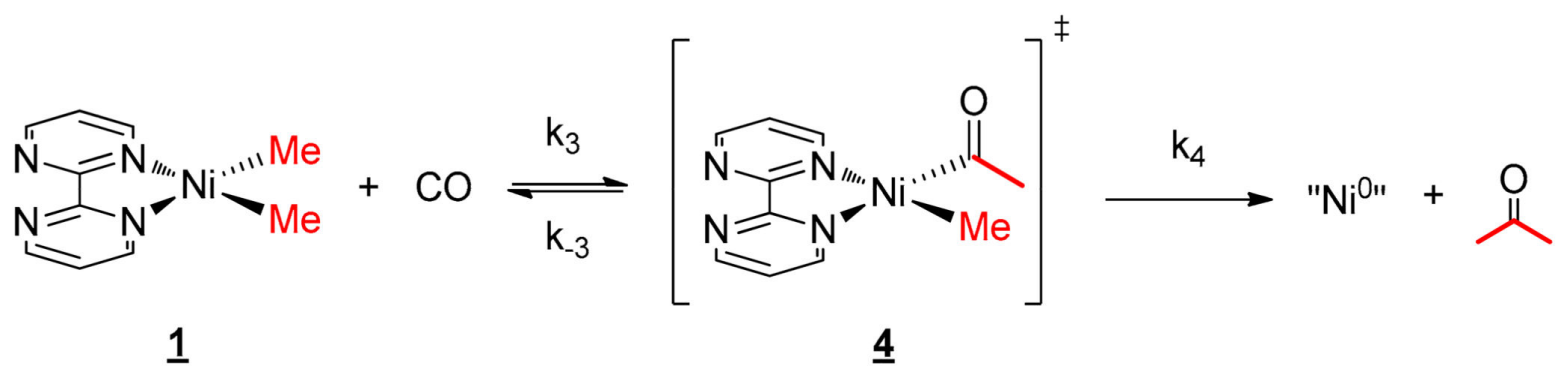
Mechanism for the reaction of **1** with CO.

The kinetics of the reaction with CO and **1** were also monitored over time by ^1^H NMR spectroscopy in order to estimate the influence of the divalent lanthanide fragment. Having discovered that the overall reaction was also following a pseudo first-order kinetics (Scheme 4), an Eyring plot was constructed with the same temperature range as the one with **2** and CO, revealing the activation parameters ∆H^≠^, and ∆S^≠^, as 17.06 kcal mol^−1^ and –16.41 cal mol^−1^ K^−1^, respectively (see Table S2 and Figure S18). The decent negative ∆S^≠^, value explains that the RDS is likely to be the reductive elimination, or more precisely, the entropically favorable rate k_4_ was much larger than k_3_ (see Supplementary Materials). Besides, the lesser ∆H^≠^, than the one of **2** indicates that there was a decreased barrier in the reaction without the organolanthanide fragment. This conclusion can also be proved with the much higher half-life time of complex **2** at 20 °C (94.7 min) compared to the one of **1** (39.0 min, see Tables S1 and S2). The comparison of these data allows the deduction that the CO migratory insertion intermediate will be much more stable in the presence of lanthanide fragments.

## 3 Materials and Methods

All reactions were performed using standard Schlenk-line techniques or in argon- or nitrogen-filled gloveboxes (MBraun, Garching, Germany). All glassware was dried at 140 °C for at least 12 h prior to use. Tetrahydrofuran (THF), THF-*d*_8_, toluene, and toluene-*d*_8_ were dried over sodium, degassed, and transferred under reduced pressure in a cold flask.

^1^H NMR spectra were recorded in 5-mm tubes adapted with a J. Young valve on Bruker AVANCE II or III-300 MHz (Bruker, Billerica, MA, USA). ^1^H chemical shifts were expressed relative to TMS (Tetramethylsilane) in ppm. Magnetic susceptibility measurements were made for all samples on powder in sealed quartz tubes at 0.5 and 20 kOe in a 7 T Cryogenic SX600 SQUID magnetometer (Cryogenic, London, UK). Diamagnetic corrections were made using Pascal’s constants. Elemental analyses were obtained from Mikroanalytisches Labor Pascher (Remagen, Germany).

(Tmeda)NiMe_2_ [41] and Cp*_2_Yb(OEt_2_) [49] complexes were synthetized according to published procedures. The bipyrimidine from TCI (Tokyo, Japan) was sublimated prior to use.

### 3.1 Synthesis of (bipym)Ni(Me)_2_ (**1**)

(tmeda)NiMe_2_ (146 mg, 0.71 mmol, 1.0 equiv.) and bipyrimidine (113 mg, 0.71 mmol, 1.0 equiv.) were respectively dissolved in cold THF (−35 °C). Transferring the bipyrimidine solution dropwise into the greenish yellow nickel solution at ambient temperature gave a dark-colored mixture after stirring for several minutes. Then, the mixture was stirred for 2 h and was stored at −35 °C in order to crystallize. Black crystalline product was obtained after one night and isolated in 73% yield (105 mg, 0.42 mmol). Yield: 73%. ^1^H NMR (300 MHz, 293 K, thf-*d*_8_): δ (ppm) = 9.32 (m, 2H, bipym), 9.12 (m, 2H, bipym), 7.67 (m, 2H, bipym), 0.06 (s, 6H, Ni–Me). ^13^C NMR (75 MHz, 273 K, thf-*d*_8_): δ (ppm) = 162.0, 156.6, 154.6, 124.4, –5.2. Anal. calcd. for C_10_H_12_N_4_Ni: C, 48.64; H, 4.90; N, 22.69; found: C, 47.00; H, 4.59; N, 21.00.

### 3.2 Synthesis of (Cp*)_2_Yb(bipym)Ni(Me)_2_ (**2**)

Cp*_2_Yb(OEt_2_) (200 mg, 0.39 mmol, 1.0 equiv.) and (bipym)NiMe_2_ (98 mg, 0.40 mmol, 1.02 equiv.) were dissolved in toluene, respectively, and cooled down to −35 °C. Transferring the green Cp*_2_Yb solution dropwise into the Nickel solution at ambient temperature gave a dark-brown mixture once the addition was finished. Then, the mixture was stored at −35 °C in order to crystallize. Dark-brown crystals were obtained after several hours, and were isolated after washing three times with *n*-pentane, in 58% yield (156 mg, 0.22 mmol). Yield: 58%. ^1^H NMR (300 MHz, 293 K, thf-*d*_8_): δ (ppm) = 246.83 (s, 2H, bipym), 15.91 (s, 6H, –Me), 9.02 (s, 2H, bipym), 6.09 (s, 30H, Cp*), –172.34 (s, 2H, bipym). Anal. calcd for C_30_H_42_N_4_NiYb: C, 52.19; H, 6.13; N, 8.11; found: C, 53.07; H, 6.06; N, 7.31.

### 3.3 Theoretical Calculations

All calculations were performed using the ORCA 4.0.0.2 software [50]. The geometry optimizations were done at three different levels of theory (PBE [51], PBE0 [52], and TPSSh [53,54]) using scalar relativistic ZORA Hamiltonian with ZORA-def2-TZVP basis set [55] and SARC/J auxiliary basis set for Coulomb fitting [56–58]. Each time, dispersion corrections were added to the functional used in the D3 framework proposed by Grimme [59] with the addition of the Becke–Johnson damping (D3BJ) [41]. Frequencies were calculated (analytically for PBE and PBE0 and numerically for TPSSh) to ensure these structures corresponded to energy minima. Single-point energy calculations starting from PBE optimized geometry were then performed in gas phase and in a toluene *continuum* with the CPCM method [60].

### 3.4 Crystal Structure Determinations

The structure resolution was accomplished using the SHELXS-97 and SHELXT [61] programs, and the refinement was done with the SHELXL program [62,63]. The structure solution and the refinement were achieved with the PLATON software [64]. Pictures of the compound structure were obtained using the MERCURY software. During the refinement steps, all atoms except hydrogen atoms were refined anisotropically. The position of the hydrogen atoms was determined using residual electronic densities. Finally, in order to obtain a complete refinement, a weighting step followed by multiple loops of refinement was done. The structures have been deposited in the CCDC with #1901938 (**1**) and 1901939 (**2**).

### 3.5 CO Migratory Insertion Studies

Reactivity tests were conducted in 5 mm NMR tubes adapted with a J. Young valve by adding CO gas directly to a degassed frozen solution of **1** or **2** and letting it react at room temperature. Kinetic analysis was performed following the ^1^H NMR resonances. The concentration of **1** was normalized by benzene residue (used as internal standard) in the deuterated solvent and complex **2** was referred to the toluene (used as internal standard), which crystalized in the cell. Integration of the NMR signals required care.

## 4 Conclusions

We successfully synthesized an original molecule with full characterizations containing reductive divalent lanthanide and reactive NiMe_2_ fragments with a redox-active bridging ligand. The strong electron correlation occurred due to the lanthanide fragment and therefore largely influenced the reactivity behavior in carbon monoxide migratory insertion. Complex **2** had a singlet ground state and a substantially populated triplet state. It was also shown that due to the electron transfer, strong spin density was on the *N*-heteroatom ligand to the reactive metal center. These considerations led to the increased stability of the CO migratory insertion step, forming an acyl-methyl intermediate. This work provides us with a new strategy for the further mechanistic study of carbonylation reactions.

## Supplementary Material

The following are available online at http://www.mdpi.com/2304-6740/7/5/58/s1. ^1^H NMR characterization (Figures S1–S5), Kinetic analysis (Figures S6–S19 and Tables S1 and S2), Magnetism (Figure S20), Crystallographic data (Figures S21–S23 and Tables S3–S6), DFT Calculation data (Figures S24 and S25; Tables S7–S12).

Supporting information
