# Supplementary material for: Reactive Heterobimetallic Complex Combining Divalent Ytterbium and Dimethyl Nickel Fragments
Source: Inorganics (Basel). Author manuscript; Available in PMC 2019 Aug 28. (PMC6713561; doi:10.3390/inorganics7050058)
Supplement: Supporting information [file EMS83981-supplement-Supporting_information.pdf]

Supplementary Materials: Reactive Heterobimetallic Complex Combining Divalent Ytterbium and Dimethyl Nickel Fragments

Ding Wang, Jules Moutet, Maxime Tricoire, Marie Cordier and Grégory Nocton

I. NMR Spectroscopy

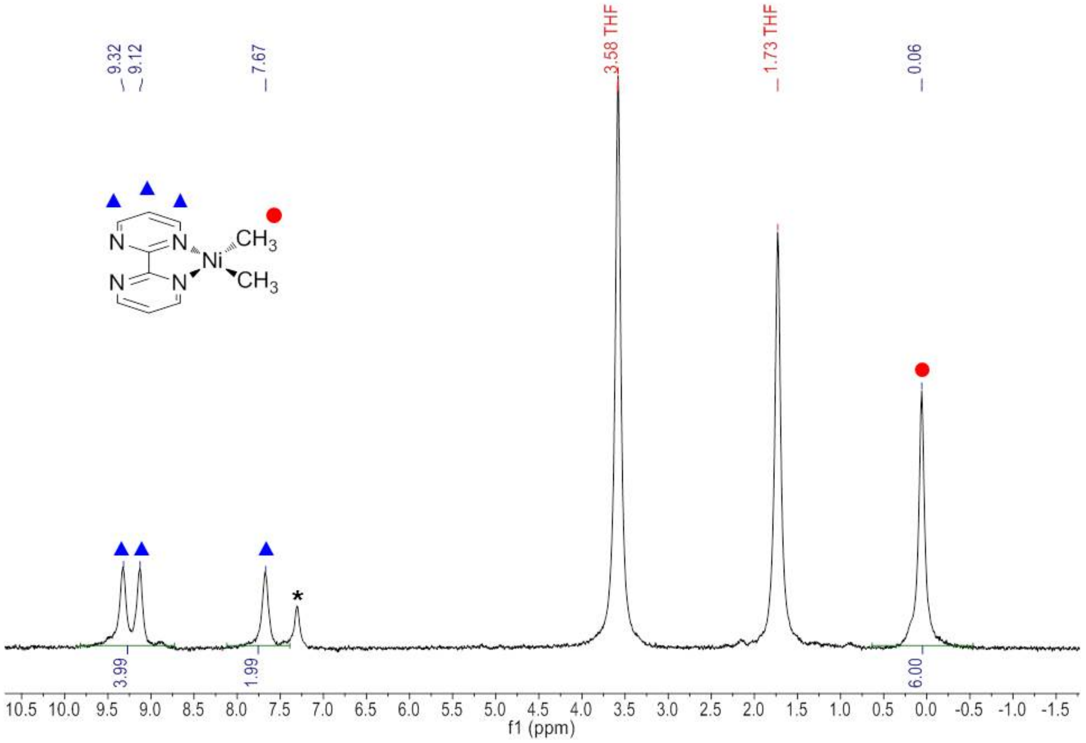

Figure S1A. ^1H NMR of (bipym)NiMe$_2$ (**1**) in thf-d$_8$ at 293 K. Benzene impurities are indicated by *.

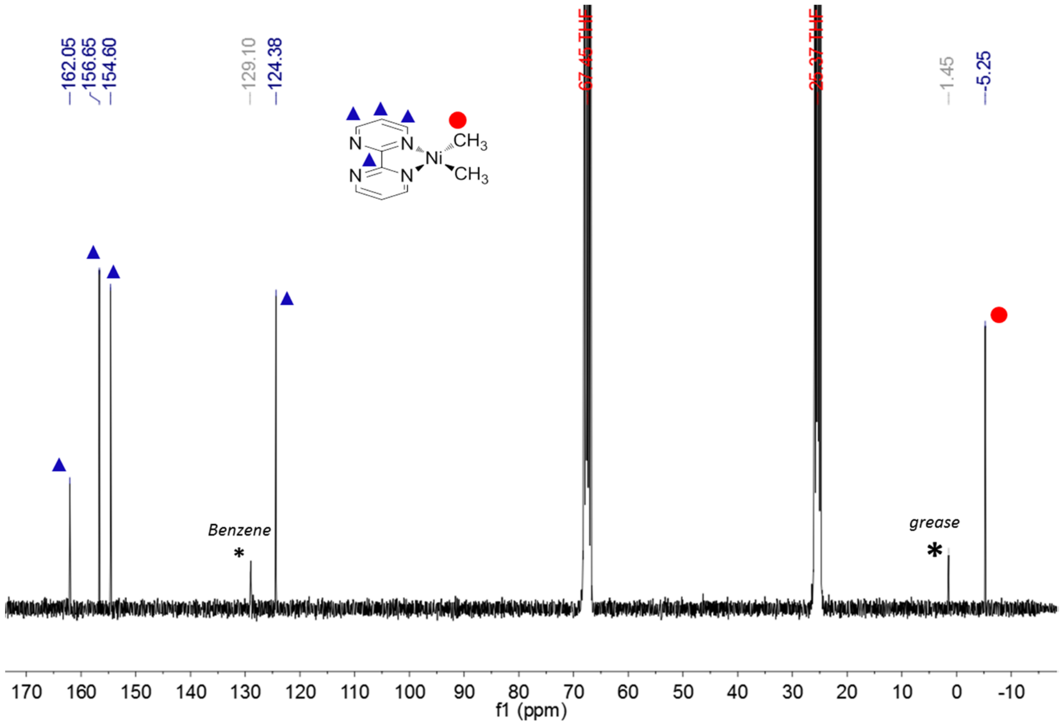

Figure S1B. ^{13}C NMR of (bipym)NiMe$_2$ (**1**) in thf-d$_8$ at 273 K. Impurities are indicated by *.

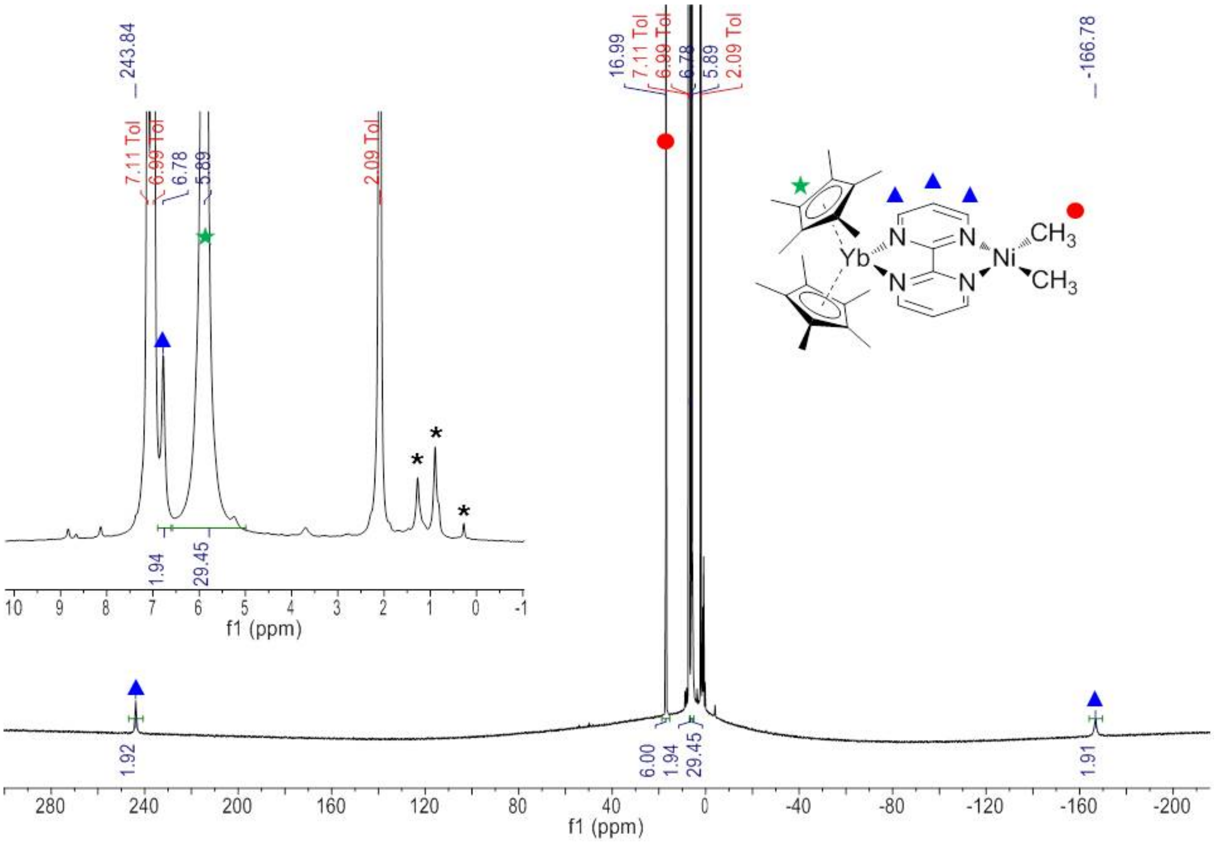

Figure S2. ^{1}H NMR of Cp*$_2$Yb(bipym)NiMe$_2$ (**2**) in tol-d$_8$ at 293 K. Pentane and grease impurities are indicated by *.

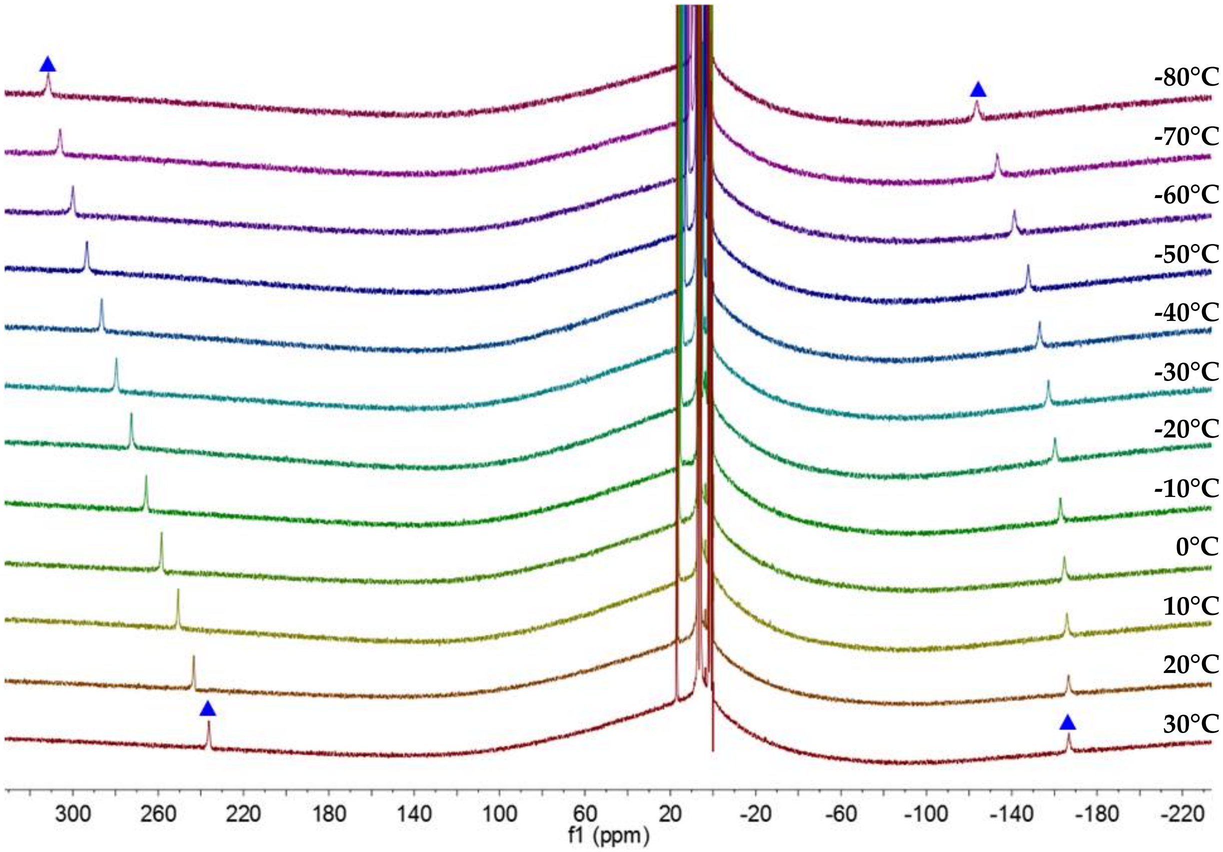

Figure S3. Variable temperature ^{1}H NMR of Cp*$_2$Yb(bipym)NiMe$_2$ (**2**) in tol-d$_8$ between 193 K and 303 K.

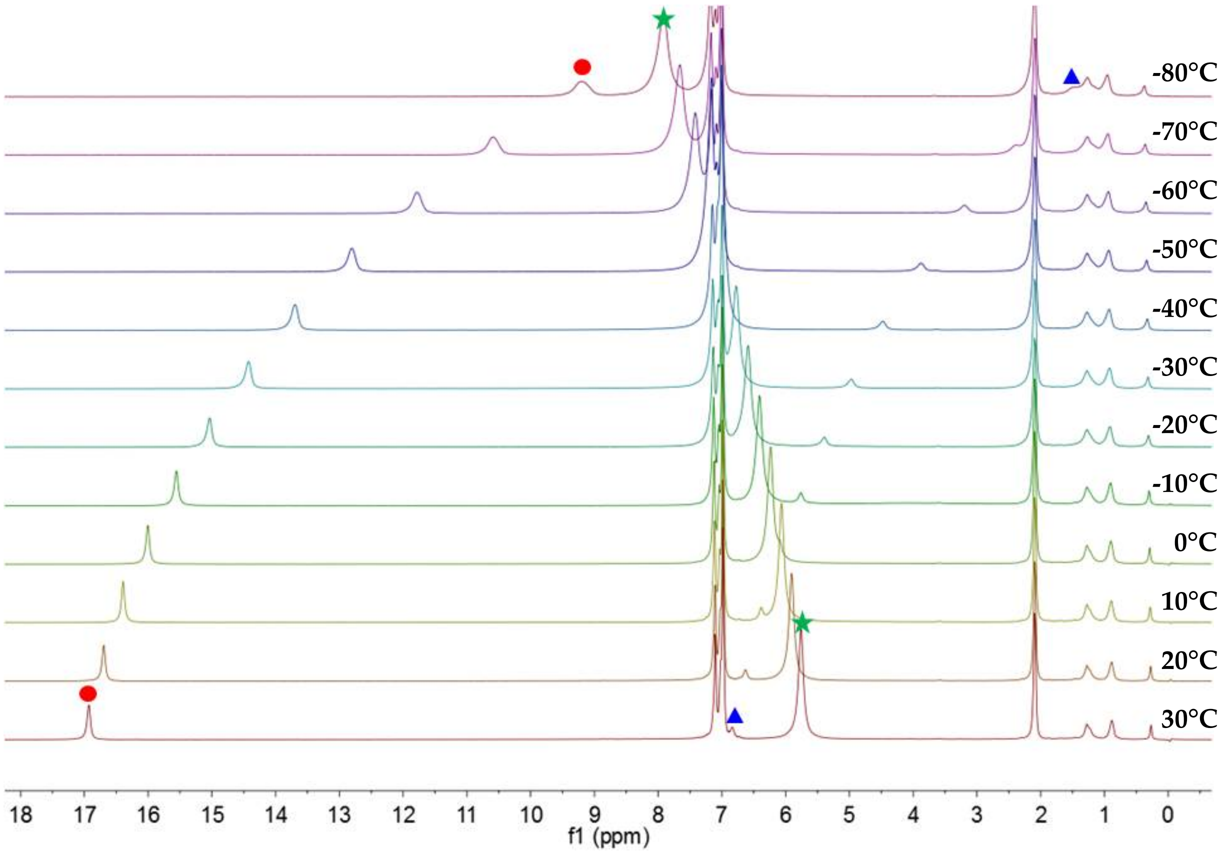

Figure S4. Variable temperature ^{1}H NMR of $Cp^*_2Yb(bipym)NiMe_2$ (**2**) in tol-d_8 between 193 K and 303 K zoom.

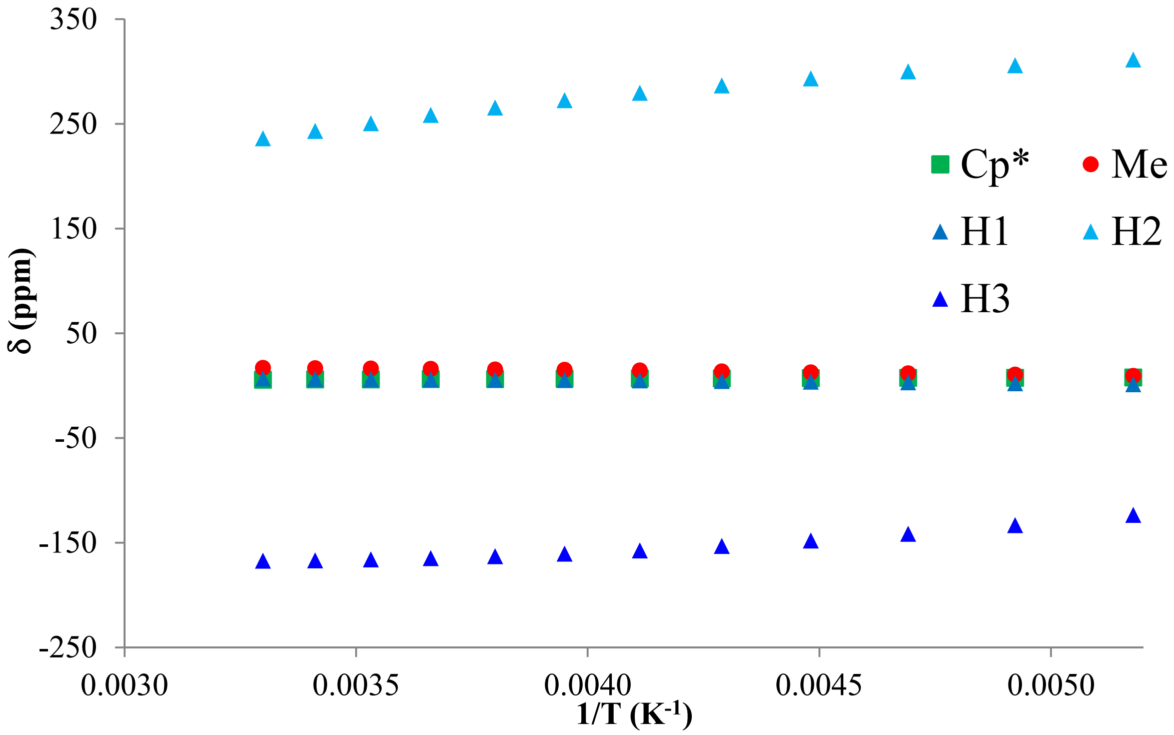

Figure S5. Variable temperature ^{1}H NMR of $Cp^*_2Yb(bipym)NiMe_2$ (**2**) in tol-d_8 plotted versus 1/T between 193 K and 303 K.

II. Kinetic analysis

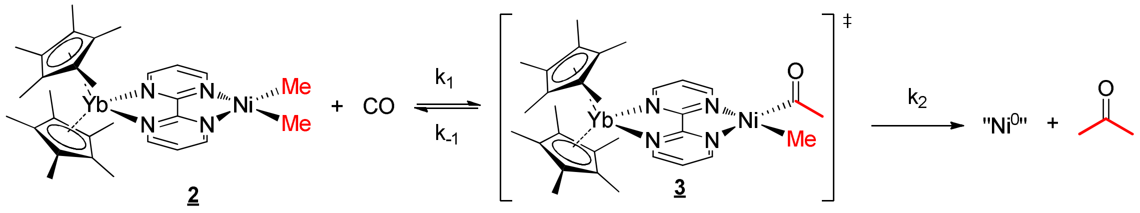

$$k_{obs} = \frac{k_1 k_2}{k_{-1} + k_2}[\mathrm{CO}]$$

Scheme S1. Mechanism for the reaction of **2** with CO

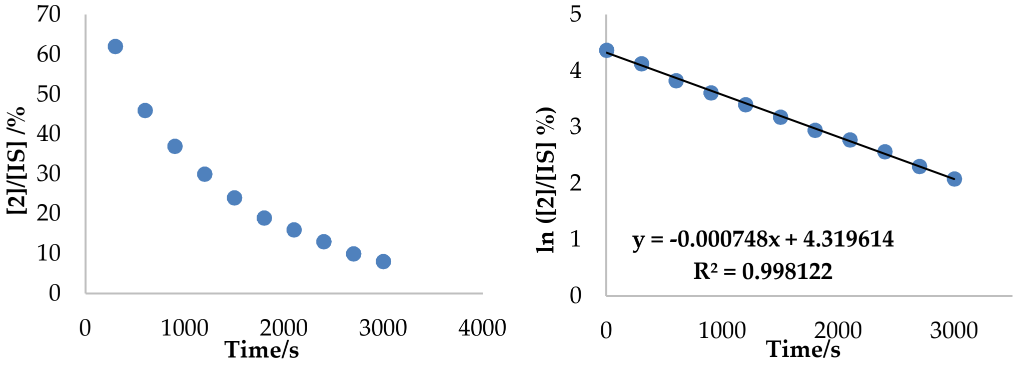

Figure S6. Kinetic data for 2 at 35 °C. $t_{1/2}$ = 926.7 s.

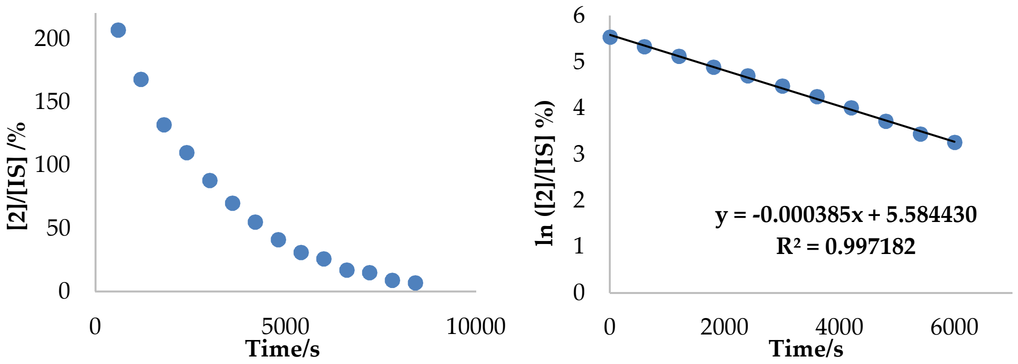

Figure S7. Kinetic data for **2** at 30 °C. $t_{1/2}$ = 1800.4 s.

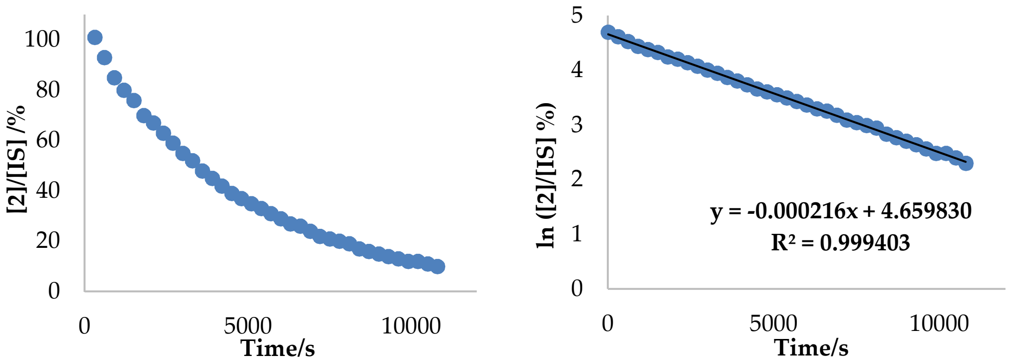

Figure S8. Kinetic data for **2** at 25 °C. $t_{1/2}$ = 3209.0 s.

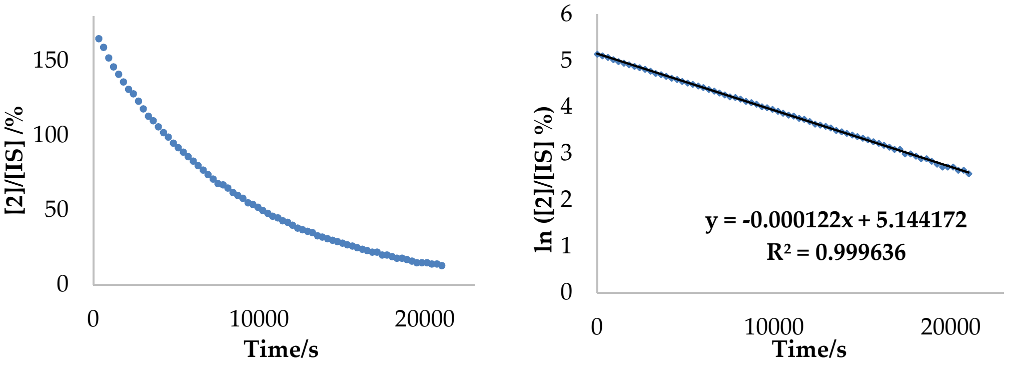

Figure S9. Kinetic data for **2** at 20 °C. $t_{1/2}$ = 5681.5 s.

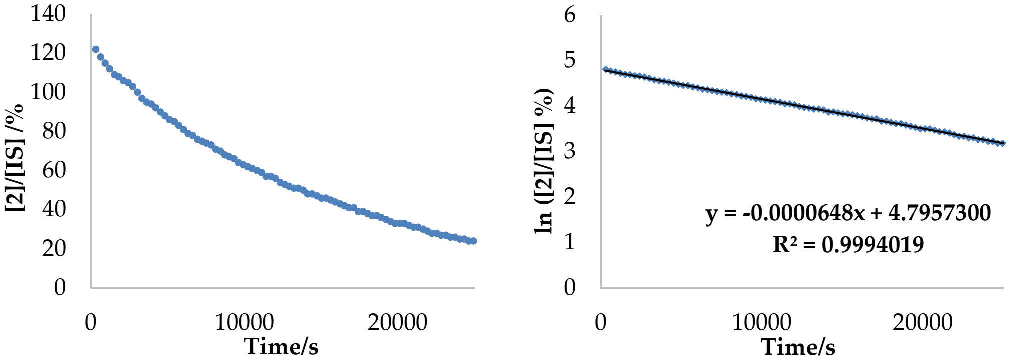

Figure S10. Kinetic data for **2** at 15 °C. $t_{1/2}$ = 10696.7 s.

Table S1. Erying Plot data for **2**.

T (°C)	T (K)	1/T (K^{-1})	k (s^{-1})	k/T (s^{-1} K^{-1})	ln (k/T)	$t_{1/2}$ (s)
35	308.15	0.003245173	0.000748	2.42739E-06	-12.928694	926.7
30	303.15	0.003298697	0.000385	1.27E-06	-13.576495	1800.4
25	298.15	0.003354016	0.000216	7.24468E-07	-14.137829	3209.0
20	293.15	0.003411223	0.000122	4.16169E-07	-14.692174	5681.5
15	288.15	0.003470415	0.0000648	2.24883E-07	-15.307686	10696.7

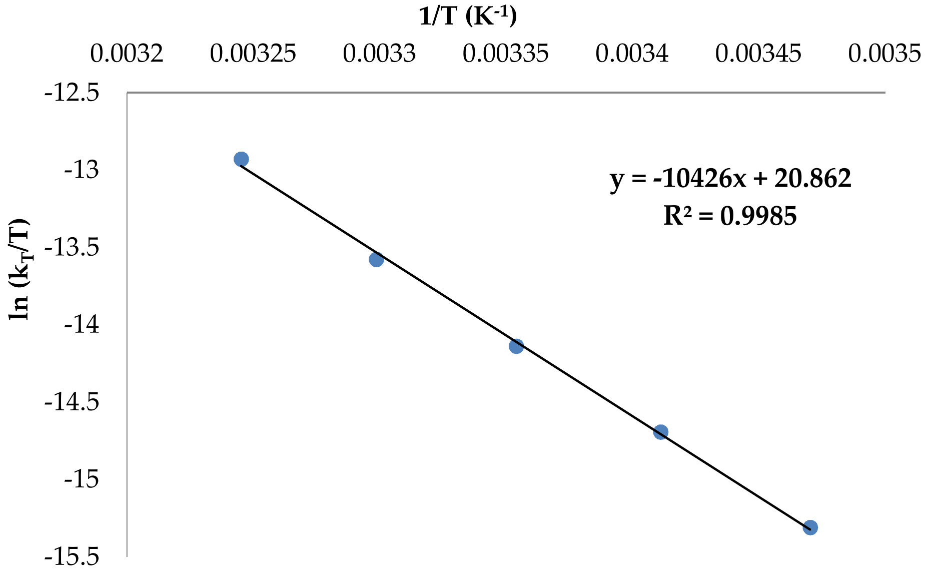

ΔH≠ = 86.68 kJ mol^{-1} (20.7 kcal.mol^{-1})
ΔS≠ = -24.09 J mol^{-1} K^{-1} (-5.75 cal.mol^{-1}.K^{-1})

Figure S11. Erying Plot and resulting ΔH≠ and ΔS≠ for **2**

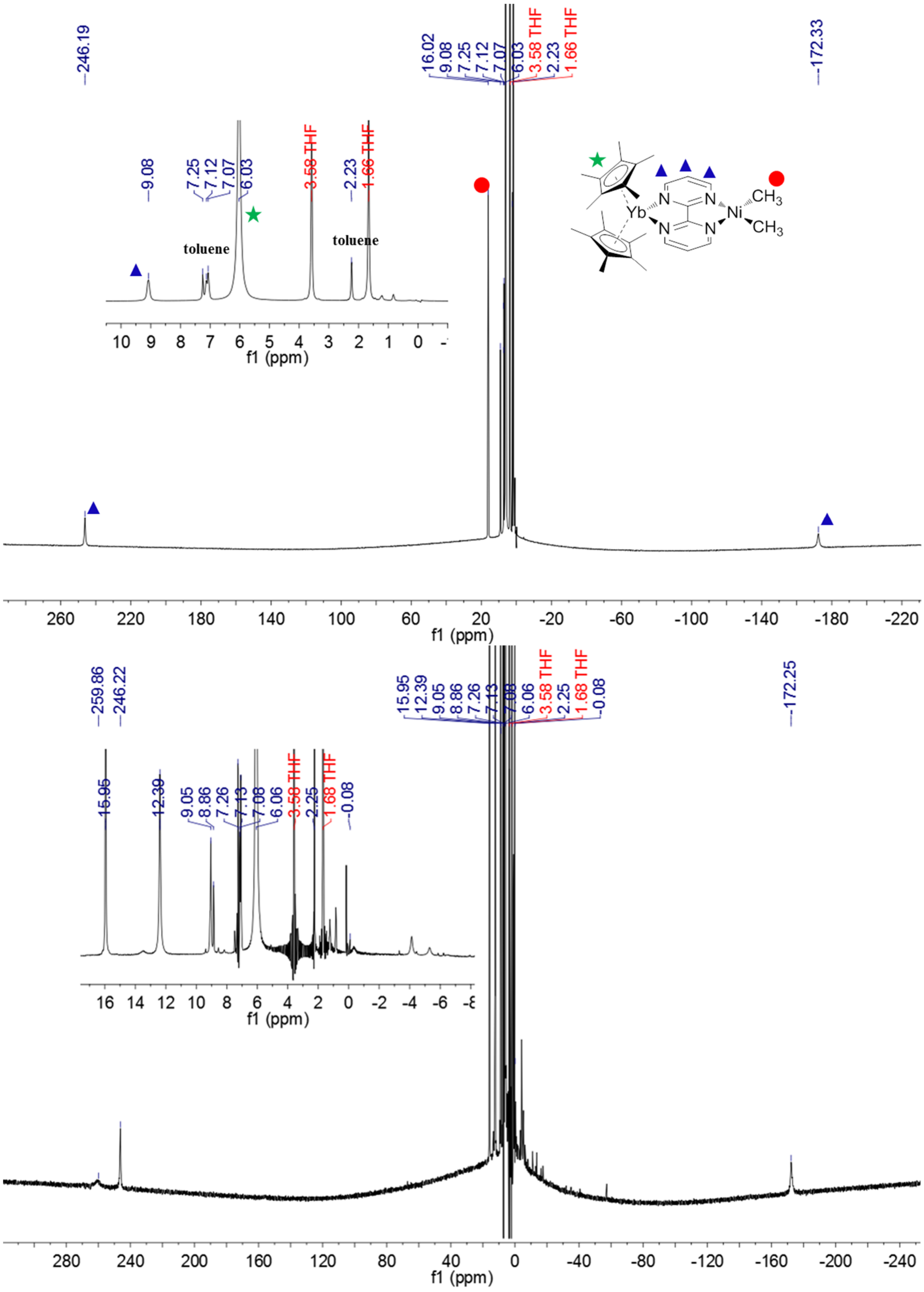

Figure S12A. ^{1}H NMR of **2** in thf-d_8 at 293 K (**a**) before addition of CO (**b**) after addition of CO (continued).

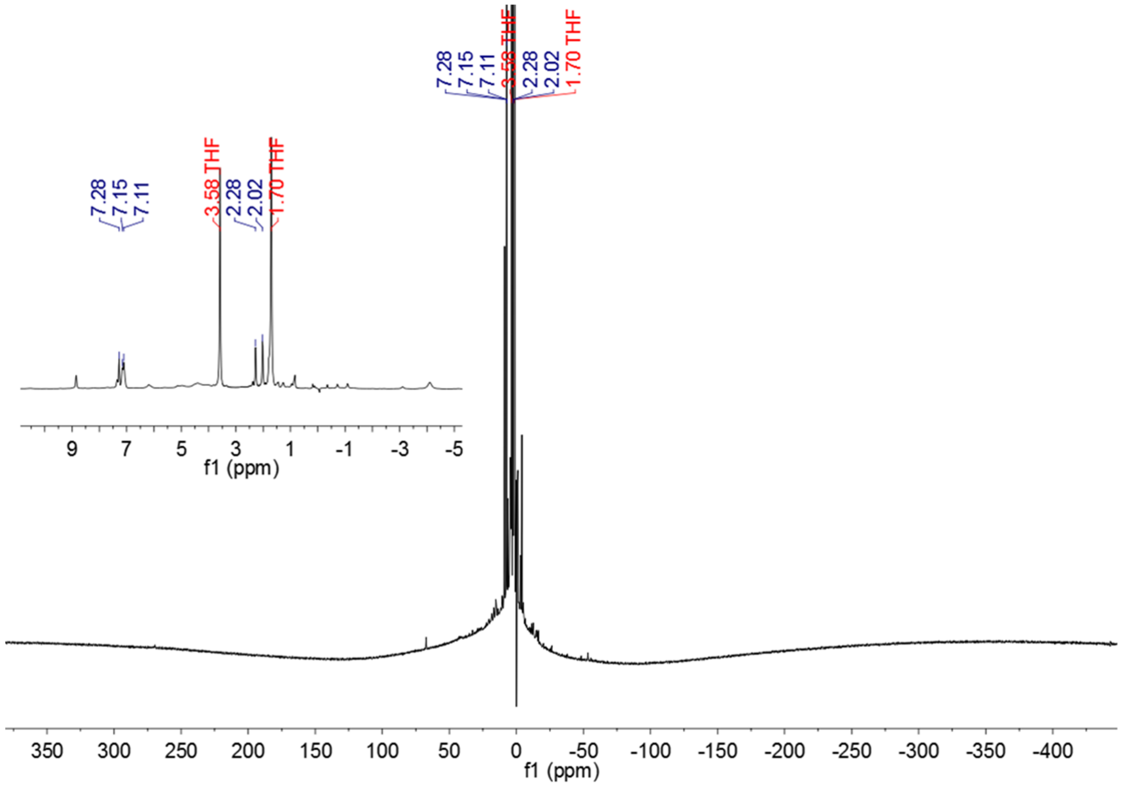

Figure S12B. ^{1}H NMR of **2** in thf-d_8 at 293 K (**c**) after the reaction with CO.

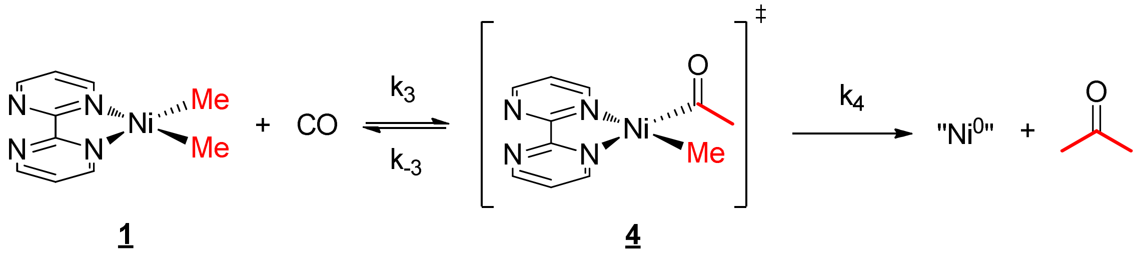

$$k_{obs} = \frac{k_3 k_4}{k_{-3} + k_4}[\mathrm{CO}]$$

Scheme S2. Mechanism for the reaction of **1** with CO.

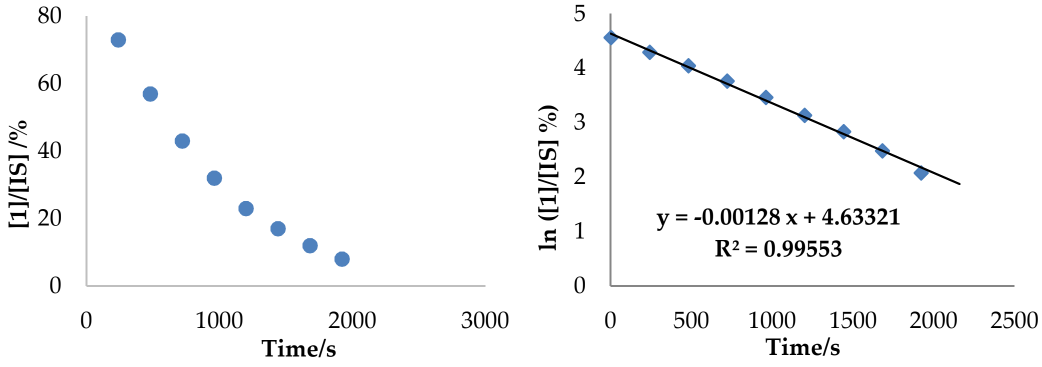

Figure S13. Kinetic data for **1** at 35 °C. $t_{1/2}$ = 541.5 s.

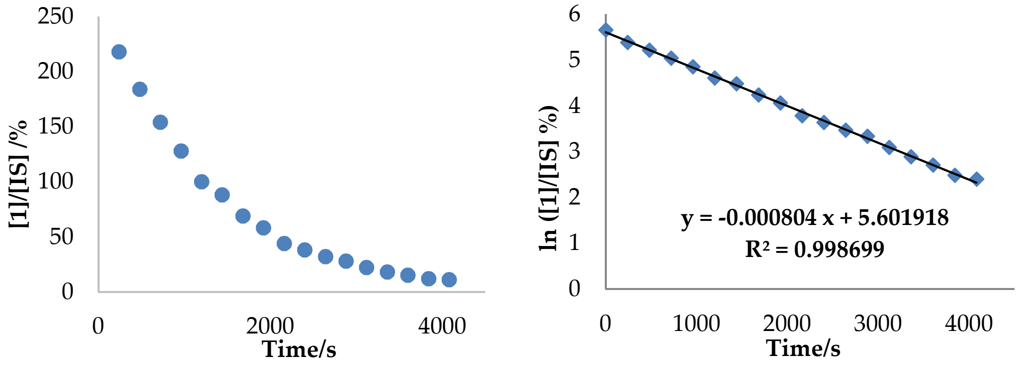

Figure S14. Kinetic data for **1** at 30 °C. $t_{1/2}$ = 862.1 s.

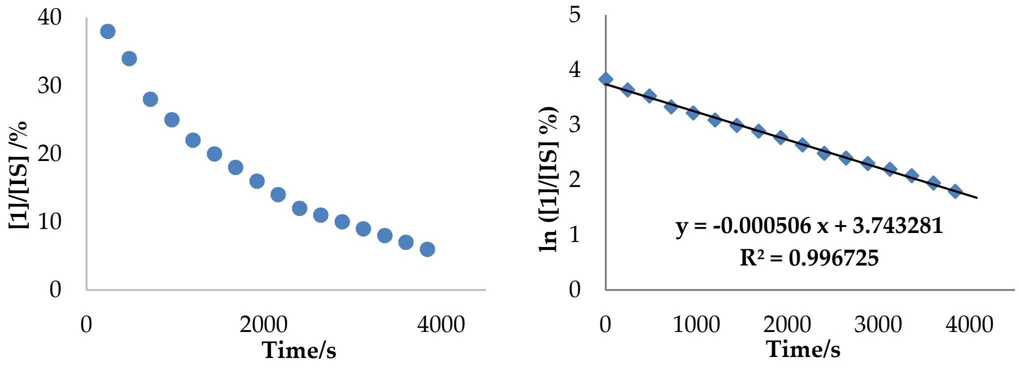

Figure S15. Kinetic data for **1** at 25 °C. $t_{1/2}$ = 1369.9 s.

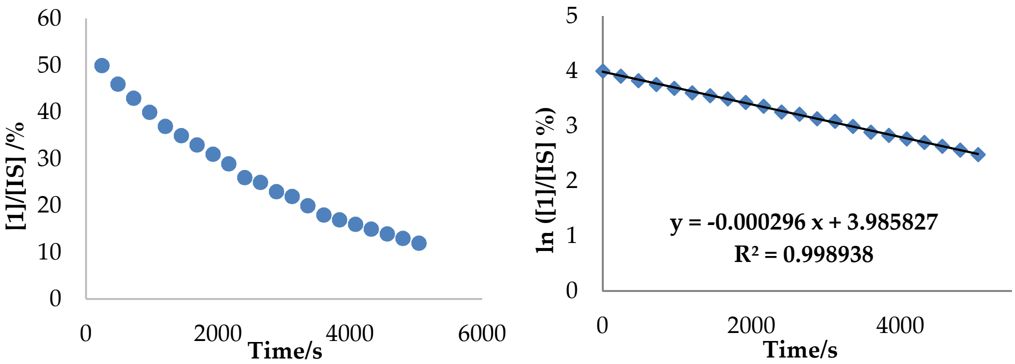

Figure S16. Kinetic data for **1** at 20 °C. $t_{1/2}$ = 2341.7 s.

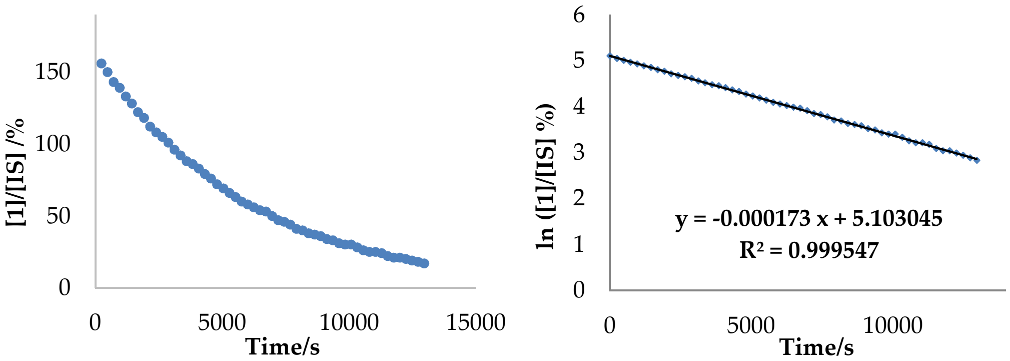

Figure S17. Kinetic data for **1** at 15 °C. $t_{1/2}$ = 4006.6 s.

Table S2. Erying Plot data for **1**.

T (°C)	T (K)	1/T (K^{-1})	k (s^{-1})	k/T (s^{-1} K^{-1})	ln (k/T)	$t_{1/2}$ (s)
35	308.15	0.003245173	0.00128	4.15382E-06	-12.391482	541.5
30	303.15	0.003298697	0.000804	2.65215E-06	-12.840139	862.1
25	298.15	0.003354016	0.000506	1.69713E-06	-13.286571	1369.9
20	293.15	0.003411223	0.000296	1.00972E-06	-13.805836	2341.7
15	288.15	0.003470415	0.000173	6.00382E-07	-14.3257	4006.6

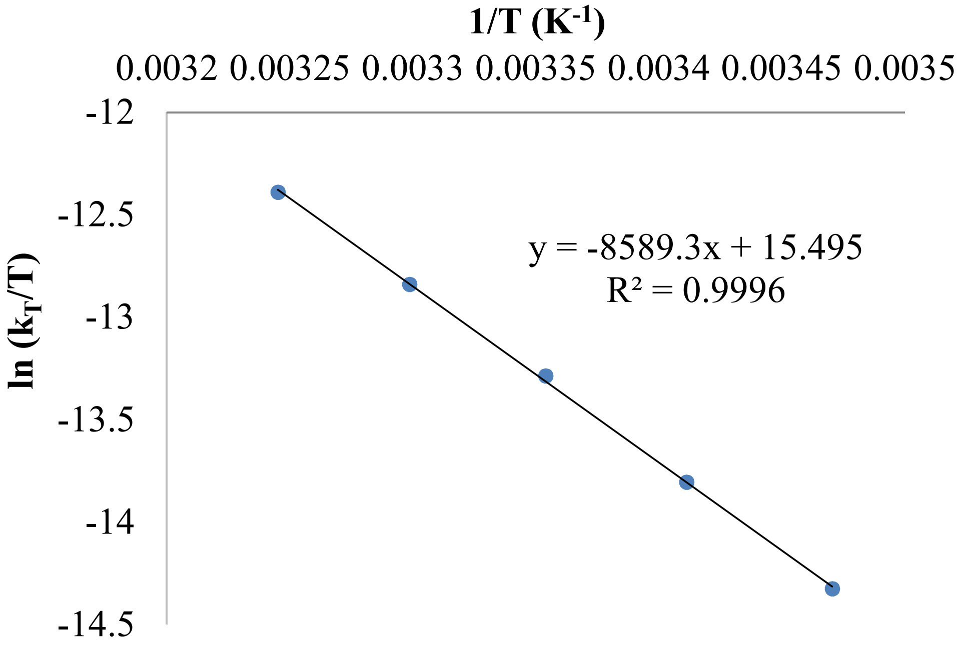

$\Delta H^{\neq}$ = 71.41 kJ mol^{-1} (17.06 $kcal.mol^{-1}$)

$\Delta S^{\neq}$ = -68.72 J mol^{-1} K^{-1} (-16.41 $cal.mol^{-1}.K^{-1}$)

Figure S18. Erying Plot and resulting $\Delta H^{\neq}$ and $\Delta S^{\neq}$ for **1.**

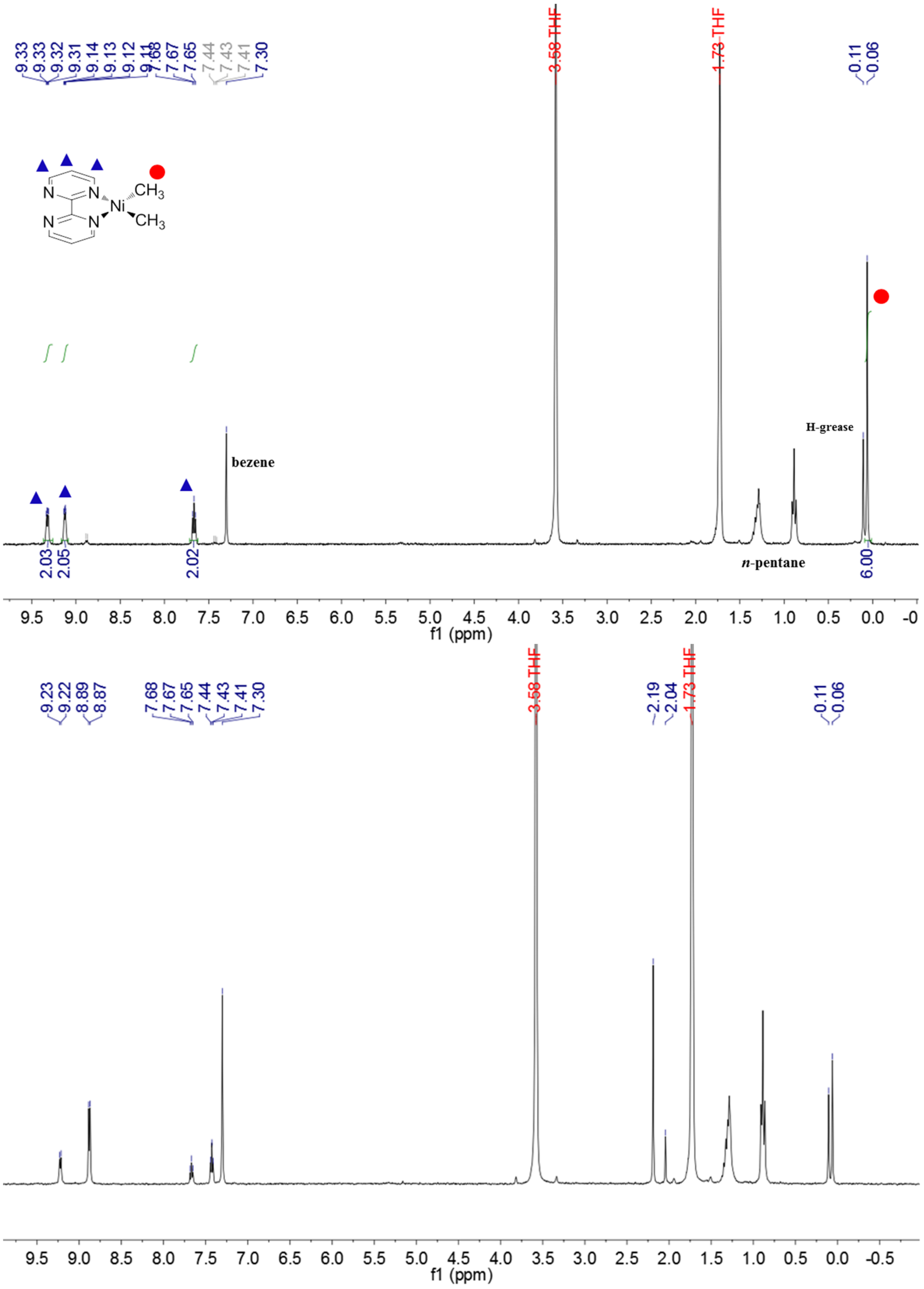

Figure S19A. ^{1}H NMR of **1** in thf-d_8 at 293 K (**a**) before addition of CO (**b**) after addition of CO (continued).

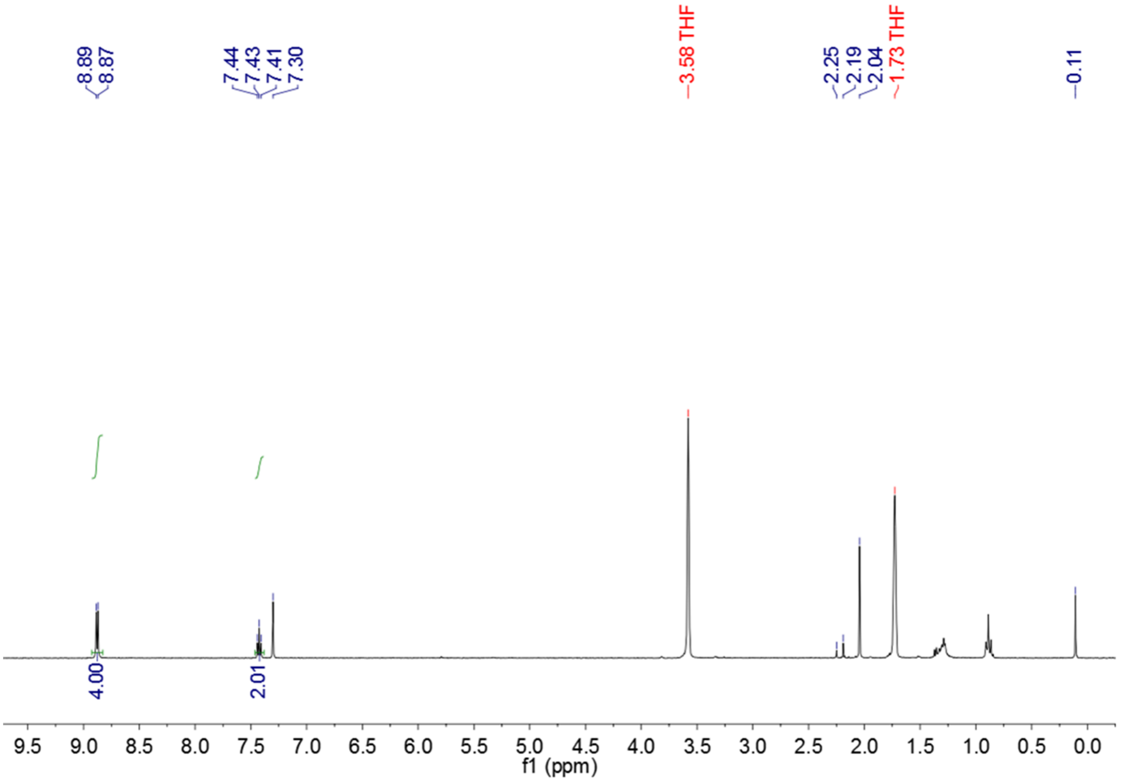

Figure S19B. ^{1}H NMR of **1** in thf-d_8 at 293 K (**c**) after the reaction with CO.

III. Magnetism

Magnetic properties of the compounds were measured on crushed crystals in sealed quartz tubes.

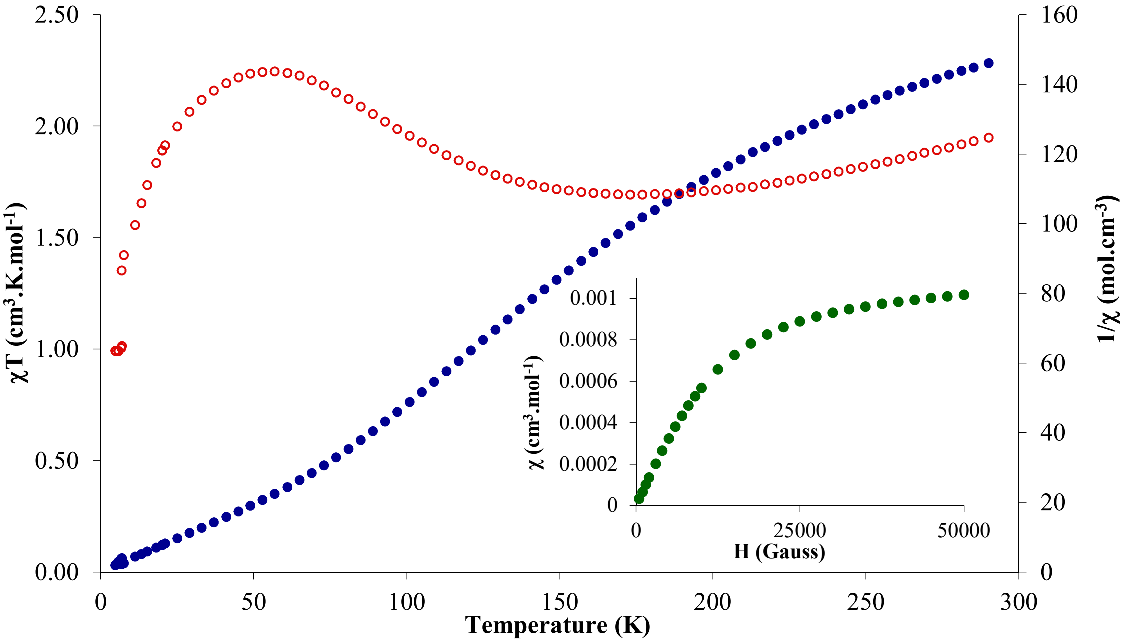

Figure S20. Temperature dependent magnetic data for **2** at 0.2T. $1/\chi$ vs T is given as unfilled red dots, χT vs T as filled blue dots. Inset show magnetization vs H at 5K.

IV. Crystallographic data

Table S3. Crystallographic data for (bipym)NiMe$_2$ (**1**) and Cp*$_2$Yb(bipym)NiMe$_2$ (**2**).

Compound	(bipym)NiMe$_2$ (1)	Cp*$_2$Yb(bipym)NiMe$_2$ (2)
Molecular formula	'C_{10} H_{12} N_4 Ni'	'C_{30} H_{42} N_4 Ni Yb, C_7 H_8'
Molecular weight	246.95	782.56
Crystal habit	black needle	red purple plate
Crystal dimensions(mm)	0.300x0.040x0.020	0.280x0.140x0.100
Crystal system	monoclinic	triclinic
Space group	C 2/c	P -1
a(Å)	9.5916(10)	9.4362(7)
b(Å)	33.666(3)	10.7558(7)
c(Å)	13.3219(13)	17.0271(12)
α(°)	90	103.204(2)
β(°)	105.255(3)	94.650(2)
γ(°)	90	93.872(2)
V(Å^3)	4150.2(7)	1670.3(2)
Z	16	2
d(g-cm^{-3})	1.581	1.556
F(000)	2048	796
m(cm^{-1})	1.839	3.377
Absorption corrections	multi-scan; 0.6496 min, 0.7456 max	multi-scan; 0.6046 min, 0.7456 max
Diffractometer	Kappa APEX II	Kappa APEX II
X-ray source	MoKa	MoKa
λ(Å)	MoKa	0.71069
Monochromator	graphite	graphite
T (K)	150.0(1)	150.0(1)
Scan mode	phi and omega scans	phi and omega scans
Maximum q	25.68	26.369
HKL ranges	-11 11 ; -40 40 ; -13 16	-11 11 ; -13 13 ; -21 21
Reflections measured	13457	26666
Unique data	3910	6781
Rint	0.0379	0.045
Reflections used	3204	6241
Criterion	I > 2(I)	I > 2σI)
Refinement type	Fsqd	Fsqd
Hydrogen atoms	constr	constr
Parameters refined	275	404
Reflections / parameter	11	15
wR2	0.0793	0.0588
Flack's parameter	0.0308	0.0236
Weights a, b	0.0391 ; 2.5054	0.0346 ; 0.1167
GoF	1.038	0.945
difference peak / hole (e Å^{-3})	0.376(0.062) / -0.244(0.062)	0.538(0.092) / -0.427(0.092)

Table S4. Average main distances (Å) and angles (°) for (bipym)NiMe$_2$ (**1**) and (Cp*)$_2$Yb(bipym)NiMe$_2$ (**2**).

Atoms	1	2
Ni-Me	1.930(3)	1.925(1)
Ni-N	1.959(2)	1.956(3)
C-C$_{bipym}$	1.482(5)	1.403(4)
Yb-N	-	2.359(1)
Yb-Cp*$_{ctr}$	-	2.31(1)
Me-Ni-Me^N-Ni-N	5.34±0.24	1.80

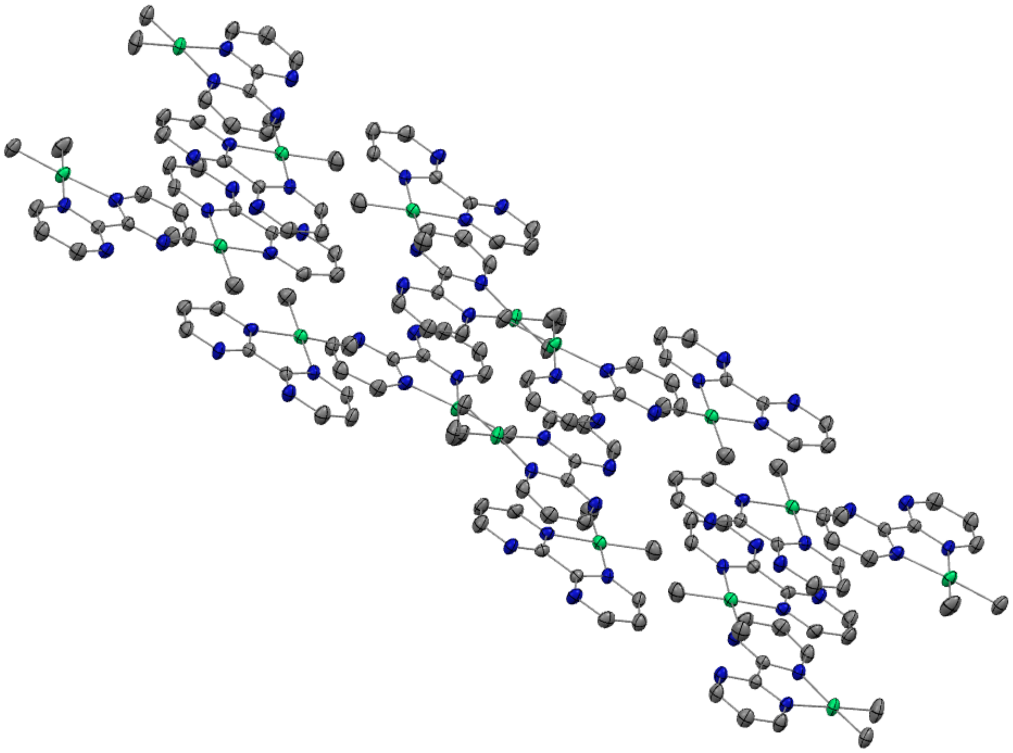

Figure S21. Packing diagram of (bipym)NiMe$_2$ (**1**). Thermal ellipsoids are at 50 % level. Carbon atoms are in grey, hydrogen atoms in grey, nitrogen atoms in blue. Hydrogen atoms are not shown for clarity.

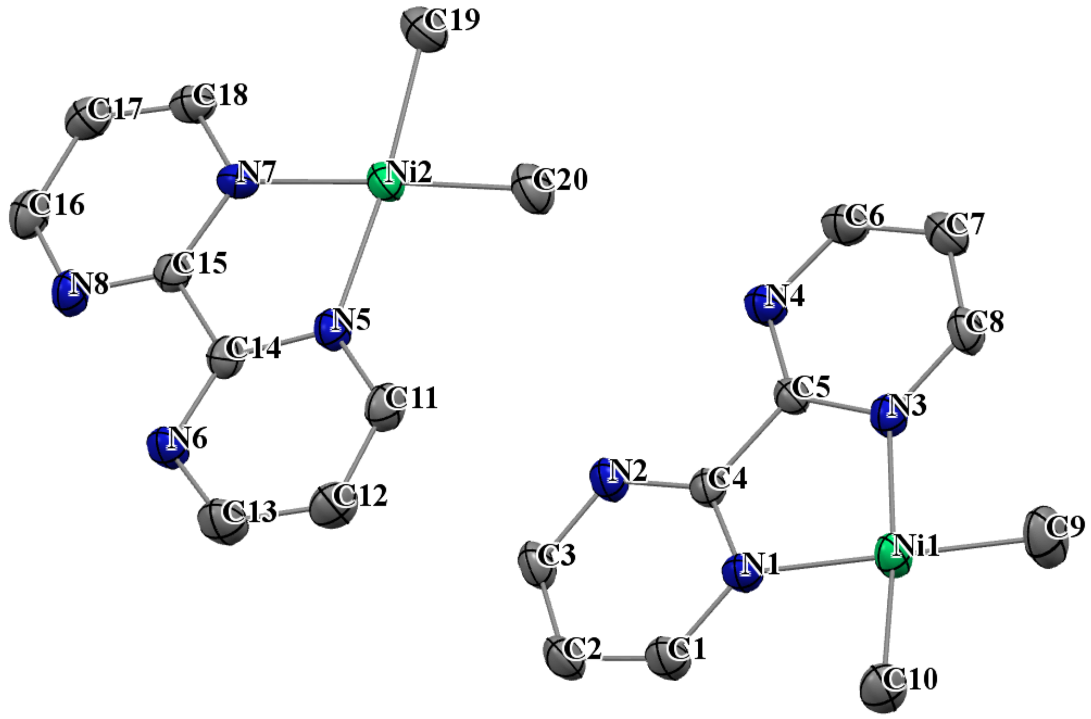

Figure S22. ORTEP asymmetric unit of (bipym)$NiMe_2$ (**1**). Thermal ellipsoids are at 50 % level. Carbon atoms are in grey, hydrogen atoms in grey, nitrogen atoms in blue. Hydrogen atoms are not shown for clarity.

Table S5. Bond lengths (Å) and angles (deg) for (bipym)$NiMe_2$ (**1**).

Ni(1)-C(9)	1.927(3)	Ni(1)-C(10)	1.930(3)
Ni(1)-N(1)	1.956(2)	Ni(1)-N(3)	1.963(2)
N(1)-C(1)	1.347(3)	N(1)-C(4)	1.358(3)
N(2)-C(4)	1.331(3)	N(2)-C(3)	1.335(3)
N(3)-C(5)	1.352(3)	N(3)-C(8)	1.356(3)
N(4)-C(5)	1.329(3)	N(4)-C(6)	1.338(3)
C(1)-C(2)	1.368(4)	C(1)-H(1)	0.9500
C(2)-C(3)	1.381(4)	C(2)-H(2)	0.9500
C(3)-H(3)	0.9500	C(4)-C(5)	1.477(3)
C(6)-C(7)	1.375(4)	C(6)-H(6)	0.9500
C(7)-C(8)	1.372(4)	C(7)-H(7)	0.9500
C(8)-H(8)	0.9500	C(9)-H(9A)	0.9800
C(9)-H(9B)	0.9800	C(9)-H(9C)	0.9800
C(10)-H(10A)	0.9800	C(10)-H(10B)	0.9800
C(10)-H(10C)	0.9800	Ni(2)-C(20)	1.927(3)
Ni(2)-C(19)	1.934(3)	Ni(2)-N(5)	1.957(2)
Ni(2)-N(7)	1.959(2)	N(5)-C(11)	1.356(3)
N(5)-C(14)	1.359(3)	N(6)-C(14)	1.319(3)
N(6)-C(13)	1.343(3)	N(7)-C(18)	1.351(3)
N(7)-C(15)	1.351(3)	N(8)-C(15)	1.328(3)
N(8)-C(16)	1.340(3)	C(11)-C(12)	1.367(4)
C(11)-H(11)	0.9500	C(12)-C(13)	1.374(4)
C(12)-H(12)	0.9500	C(13)-H(13)	0.9500
C(14)-C(15)	1.487(3)	C(16)-C(17)	1.376(3)
C(16)-H(16)	0.9500	C(17)-C(18)	1.373(3)
C(17)-H(17)	0.9500	C(18)-H(18)	0.9500

C(19)-H(19A)	0.9800	C(19)-H(19B)	0.9800
C(19)-H(19C)	0.9800	C(20)-H(20A)	0.9800
C(20)-H(20B)	0.9800	C(20)-H(20C)	0.9800

C(9)-Ni(1)-C(10)	87.6(1)	C(9)-Ni(1)-N(1)	176.5(1)
C(10)-Ni(1)-N(1)	94.6(1)	C(9)-Ni(1)-N(3)	96.3(1)
C(10)-Ni(1)-N(3)	174.1(1)	N(1)-Ni(1)-N(3)	81.73(8)
C(1)-N(1)-C(4)	115.0(2)	C(1)-N(1)-Ni(1)	129.6(2)
C(4)-N(1)-Ni(1)	115.3(2)	C(4)-N(2)-C(3)	116.0(2)
C(5)-N(3)-C(8)	115.1(2)	C(5)-N(3)-Ni(1)	115.6(2)
C(8)-N(3)-Ni(1)	129.1(2)	C(5)-N(4)-C(6)	115.6(2)
N(1)-C(1)-C(2)	122.6(2)	N(1)-C(1)-H(1)	118.7
C(2)-C(1)-H(1)	118.7	C(1)-C(2)-C(3)	117.3(2)
C(1)-C(2)-H(2)	121.3	C(3)-C(2)-H(2)	121.3
N(2)-C(3)-C(2)	122.4(2)	N(2)-C(3)-H(3)	118.8
C(2)-C(3)-H(3)	118.8	N(2)-C(4)-N(1)	126.7(2)
N(2)-C(4)-C(5)	119.5(2)	N(1)-C(4)-C(5)	113.9(2)
N(4)-C(5)-N(3)	127.1(2)	N(4)-C(5)-C(4)	119.5(2)
N(3)-C(5)-C(4)	113.4(2)	N(4)-C(6)-C(7)	122.8(2)
N(4)-C(6)-H(6)	118.6	C(7)-C(6)-H(6)	118.6
C(8)-C(7)-C(6)	117.4(2)	C(8)-C(7)-H(7)	121.3
C(6)-C(7)-H(7)	121.3	N(3)-C(8)-C(7)	122.0(2)
N(3)-C(8)-H(8)	119.0	C(7)-C(8)-H(8)	119.0
Ni(1)-C(9)-H(9A)	109.5	Ni(1)-C(9)-H(9B)	109.5
H(9A)-C(9)-H(9B)	109.5	Ni(1)-C(9)-H(9C)	109.5
H(9A)-C(9)-H(9C)	109.5	H(9B)-C(9)-H(9C)	109.5
Ni(1)-C(10)-H(10A)	109.5	Ni(1)-C(10)-H(10B)	109.5
H(10A)-C(10)-H(10B)	109.5	Ni(1)-C(10)-H(10C)	109.5
H(10A)-C(10)-H(10C)	109.5	H(10B)-C(10)-H(10C)	109.5
C(20)-Ni(2)-C(19)	87.0(1)	C(20)-Ni(2)-N(5)	96.7(1)
C(19)-Ni(2)-N(5)	174.3(1)	C(20)-Ni(2)-N(7)	177.4(1)
C(19)-Ni(2)-N(7)	94.7(1)	N(5)-Ni(2)-N(7)	81.82(8)
C(11)-N(5)-C(14)	114.4(2)	C(11)-N(5)-Ni(2)	129.8(2)
C(14)-N(5)-Ni(2)	115.7(2)	C(14)-N(6)-C(13)	116.0(2)
C(18)-N(7)-C(15)	115.1(2)	C(18)-N(7)-Ni(2)	129.4(2)
C(15)-N(7)-Ni(2)	115.5(2)	C(15)-N(8)-C(16)	115.5(2)
N(5)-C(11)-C(12)	122.3(2)	N(5)-C(11)-H(11)	118.9
C(12)-C(11)-H(11)	118.9	C(11)-C(12)-C(13)	117.9(2)
C(11)-C(12)-H(12)	121.0	C(13)-C(12)-H(12)	121.0
N(6)-C(13)-C(12)	122.0(2)	N(6)-C(13)-H(13)	119.0
C(12)-C(13)-H(13)	119.0	N(6)-C(14)-N(5)	127.3(2)
N(6)-C(14)-C(15)	119.8(2)	N(5)-C(14)-C(15)	112.9(2)
N(8)-C(15)-N(7)	127.1(2)	N(8)-C(15)-C(14)	119.0(2)
N(7)-C(15)-C(14)	113.9(2)	N(8)-C(16)-C(17)	122.8(2)
N(8)-C(16)-H(16)	118.6	C(17)-C(16)-H(16)	118.6
C(18)-C(17)-C(16)	117.1(2)	C(18)-C(17)-H(17)	121.4
C(16)-C(17)-H(17)	121.4	N(7)-C(18)-C(17)	122.3(2)
N(7)-C(18)-H(18)	118.8	C(17)-C(18)-H(18)	118.8
Ni(2)-C(19)-H(19A)	109.5	Ni(2)-C(19)-H(19B)	109.5
H(19A)-C(19)-H(19B)	109.5	Ni(2)-C(19)-H(19C)	109.5
H(19A)-C(19)-H(19C)	109.5	H(19B)-C(19)-H(19C)	109.5
Ni(2)-C(20)-H(20A)	109.5	Ni(2)-C(20)-H(20B)	109.5
H(20A)-C(20)-H(20B)	109.5	Ni(2)-C(20)-H(20C)	109.5
H(20A)-C(20)-H(20C)	109.5	H(20B)-C(20)-H(20C)	109.5

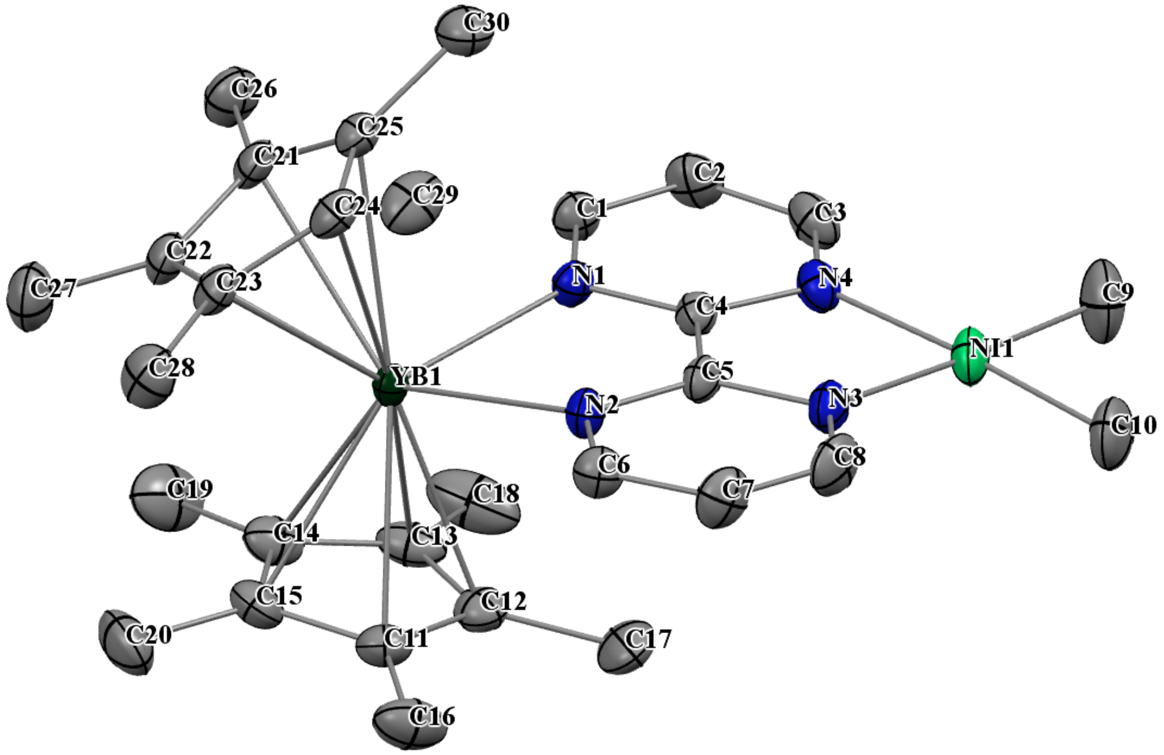

Figure S23. ORTEP asymmetric unit of Cp*$_2$Yb(bipym)NiMe$_2$ (**2**). Thermal ellipsoids are at 50 % level. Carbon atoms are in grey, hydrogen atoms in grey, nitrogen atoms in blue. Hydrogen atoms and solvent molecules are not shown for clarity.

Table S6. Bond lengths (Å) and angles (deg) for Cp*$_2$Yb(bipym)NiMe$_2$ (**2**).

Yb(1)-N(2)	2.358(2)	Yb(1)-N(1)	2.359(2)
Yb(1)-C(21**)**	2.585(3)	Yb(1)-C(11)	2.592(3)
Yb(1)-C(15)	2.598(3)	Yb(1)-C(22)	2.600(3)
Yb(1)-C(12)	2.604(3)	Yb(1)-C(13)	2.605(3)
Yb(1)-C(14)	2.606(3)	Yb(1)-C(25)	2.617(3)
Yb(1)-C(23)	2.620(3)	Yb(1)-C(24)	2.627(3)
Ni(1)-C(9)	1.924(3)	Ni(1)-C(10)	1.925(3)
Ni(1)-N(3)	1.953(3)	Ni(1)-N(4)	1.959(2)
N(1)-C(1)	1.339(3)	N(1)-C(4)	1.382(4)
N(2)-C(6)	1.340(3)	N(2)-C(5)	1.373(4)
N(3)-C(8)	1.338(4)	N(3)-C(5)	1.372(4)
N(4)-C(3)	1.320(4)	N(4)-C(4)	1.378(4)
C(1)-C(2)	1.372(4	C(1)-H(1)	0.9500
C(2)-C(3)	1.390(4)	C(2)-H(2)	0.9500
C(3)-H(3)	0.9500	C(4)-C(5)	1.403(4)
C(6)-C(7)	1.378(4)	C(6)-H(6)	0.9500
C(7)-C(8)	1.376(5)	C(7)-H(7)	0.9500
C(8)-H(8)	0.9500	C(9)-H(9A)	0.9800
C(9)-H(9B)	0.9800	C(9)-H(9C)	0.9800
C(10)-H(10A)	0.9800	C(10)-H(10B)	0.9800
C(10)-H(10C)	0.9800	C(11)-C(15)	1.405(4)
C(11)-C(12)	1.419(4)	C(11)-C(16)	1.501(4)
C(12)-C(13)	1.421(4)	C(12)-C(17)	1.492(4)
C(13)-C(14)	1.407(5)	C(13)-C(18)	1.504(4)
C(14)-C(15)	1.426(4)	C(14)-C(19)	1.499(4)

C(15)-C(20)	1.504(4)	C(16)-H(16A)	0.9800
C(16)-H(16B)	0.9800	C(16)-H(16C)	0.9800
C(17)-H(17A)	0.9800	C(17)-H(17B)	0.9800
C(17)-H(17C)	0.9800	C(18)-H(18A)	0.9800
C(18)-H(18B)	0.9800	C(18)-H(18C)	0.9800
C(19)-H(19A)	0.9800	C(19)-H(19B)	0.9800
C(19)-H(19C)	0.9800	C(20)-H(20A)	0.9800
C(20)-H(20B)	0.9800	C(20)-H(20C)	0.9800
C(21)-C(25)	1.409(4)	C(21)-C(22)	1.413(4)
C(21)-C(26)	1.507(4)	C(22)-C(23)	1.421(4)
C(22)-C(27)	1.505(4)	C(23)-C(24)	1.410(4)
C(23)-C(28)	1.506(4)	C(24)-C(25)	1.424(4)
C(24)-C(29)	1.496(4)	C(25)-C(30)	1.492(4)
C(26)-H(26A)	0.9800	C(26)-H(26B)	0.9800
C(26)-H(26C)	0.9800	C(27)-H(27A)	0.9800
C(27)-H(27B)	0.9800	C(27)-H(27C)	0.9800
C(28)-H(28A)	0.9800	C(28)-H(28B)	0.9800
C(28)-H(28C)	0.9800	C(29)-H(29A)	0.9800
C(29)-H(29B)	0.9800	C(29)-H(29C)	0.9800
C(30)-H(30A)	0.9800	C(30)-H(30B)	0.9800
C(30)-H(30C)	0.9800	C(31)-C(32)	1.3900
C(31)-C(36)	1.3900	C(31)-H(31)	0.9500
C(32)-C(33)	1.3900	C(32)-H(32)	0.9500
C(33)-C(34)	1.3900	C(33)-H(33)	0.9500
C(34)-C(35)	1.3900	C(34)-H(34)	0.9500
C(35)-C(36)	1.3900	C(35)-H(35)	0.9500
C(36)-C(37)	1.45(1)	C(37)-H(37A)	0.9800
C(37)-H(37B)	0.9800	C(37)-H(37C)	0.9800
C(38)-C(44)	1.06(1)	C(38)-C(39)	1.3900
C(38)-C(43)	1.3900	C(39)-C(40)	1.3900
C(39)-H(39)	0.9500	C(40)-C(41)	1.3900
C(40)-H(40)	0.9500	C(41)-C(42)	1.3900
C(41)-H(41)	0.9500	C(42)-C(43)	1.3900
C(42)-H(42)	0.9500	C(43)-H(43)	0.9500
C(44)-H(44A)	0.9800	C(44)-H(44B)	0.9800
C(44)-H(44C)	0.9800		

N(2)-Yb(1)-N(1)	71.94(8)	N(2)-Yb(1)-C(21)	124.5(1)
N(1)-Yb(1)-C(21)	90.59(8)	N(2)-Yb(1)-C(11)	84.6(1)
N(1)-Yb(1)-C(11)	116.2(1)	C(21)-Yb(1)-C(11)	146.6(1)
N(2)-Yb(1)-C(15)	115.0(1)	N(1)-Yb(1)-C(15)	131.6(1)
C(21)-Yb(1)-C(15)	115.6(1)	C(11)-Yb(1)-C(15)	31.4(1)
N(2)-Yb(1)-C(22)	132.0(1)	N(1)-Yb(1)-C(22)	122.2(1)
C(21)-Yb(1)-C(22)	31.6(1)	C(11)-Yb(1)-C(22)	117.6(1)
C(15)-Yb(1)-C(22)	89.8(1)	N(2)-Yb(1)-C(12)	80.3(1)
N(1)-Yb(1)-C(12)	85.3(1)	C(21)-Yb(1)-C(12)	152.0(1)
C(11)-Yb(1)-C(12)	31.7(1)	C(15)-Yb(1)-C(12)	52.2(1)
C(22)-Yb(1)-C(12)	140.7(1)	N(2)-Yb(1)-C(13)	107.9(1)
N(1)-Yb(1)-C(13)	79.7(1)	C(21)-Yb(1)-C(13)	120.4(1)
C(11)-Yb(1)-C(13)	52.3(1)	C(15)-Yb(1)-C(13)	52.1(1)
C(22)-Yb(1)-C(13)	119.5(1)	C(12)-Yb(1)-C(13)	31.6(1)
N(2)-Yb(1)-C(14)	132.1(1)	N(1)-Yb(1)-C(14)	106.1(1)
C(21)-Yb(1)-C(14)	103.1(1)	C(11)-Yb(1)-C(14)	52.4(1)
C(15)-Yb(1)-C(14)	31.8(1)	C(22)-Yb(1)-C(14)	90.8(1)
C(12)-Yb(1)-C(14)	52.3(1)	C(13)-Yb(1)-C(14)	31.3(1)
N(2)-Yb(1)-C(25)	93.1(1)	N(1)-Yb(1)-C(25)	80.2(1)
C(21)-Yb(1)-C(25)	31.4(1)	C(11)-Yb(1)-C(25)	161.4(1)

C(15)-Yb(1)-C(25)	141.7(1)	C(22)-Yb(1)-C(25)	52.0(1)
C(12)-Yb(1)-C(25)	165.3(1)	C(13)-Yb(1)-C(25)	144.6(1)
C(14)-Yb(1)-C(25)	134.5(1)	N(2)-Yb(1)-C(23)	102.5(1)
N(1)-Yb(1)-C(23)	131.9(1)	C(21)-Yb(1)-C(23)	52.3(1)
C(11)-Yb(1)-C(23)	110.4(1)	C(15)-Yb(1)-C(23)	94.8(1)
C(22)-Yb(1)-C(23)	31.6(1)	C(12)-Yb(1)-C(23)	142.1(1)
C(13)-Yb(1)-C(23)	142.2(1)	C(14)-Yb(1)-C(23)	111.0(1)
C(25)-Yb(1)-C(23)	52.0(1)	N(2)-Yb(1)-C(24)	80.8(1)
N(1)-Yb(1)-C(24)	103.7(1)	C(21)-Yb(1)-C(24)	52.1(1)
C(11)-Yb(1)-C(24)	130.4(1)	C(15)-Yb(1)-C(24)	124.6(1)
C(22)-Yb(1)-C(24)	51.9(1)	C(12)-Yb(1)-C(24)	155.4(1)
C(13)-Yb(1)-C(24)	171.3(1)	C(14)-Yb(1)-C(24)	141.1(1)
C(25)-Yb(1)-C(24)	31.5(1)	C(23)-Yb(1)-C(24)	31.2(1)
C(9)-Ni(1)-C(10)	89.3(2)	C(9)-Ni(1)-N(3)	176.9(1)
C(10)-Ni(1)-N(3)	93.7(1)	C(9)-Ni(1)-N(4)	93.9(1)
C(10)-Ni(1)-N(4)	176.4(1)	N(3)-Ni(1)-N(4)	83.1(1)
C(1)-N(1)-C(4)	115.9(2)	C(1)-N(1)-Yb(1)	130.4(2)
C(4)-N(1)-Yb(1)	113.3(2)	C(6)-N(2)-C(5)	116.4(2)
C(6)-N(2)-Yb(1)	129.4(2)	C(5)-N(2)-Yb(1)	113.6(2)
C(8)-N(3)-C(5)	115.6(3)	C(8)-N(3)-Ni(1)	131.4(2)
C(5)-N(3)-Ni(1)	113.0(2)	C(3)-N(4)-C(4)	116.2(3)
C(3)-N(4)-Ni(1)	131.5(2)	C(4)-N(4)-Ni(1)	112.3(2)
N(1)-C(1)-C(2)	122.7(3)	N(1)-C(1)-H(1)	118.6
C(2)-C(1)-H(1)	118.6	C(1)-C(2)-C(3)	117.7(3)
C(1)-C(2)-H(2)	121.2	C(3)-C(2)-H(2)	121.2
N(4)-C(3)-C(2)	123.0(3)	N(4)-C(3)-H(3)	118.5
C(2)-C(3)-H(3)	118.5	N(4)-C(4)-N(1)	124.4(2)
N(4)-C(4)-C(5)	115.9(3)	N(1)-C(4)-C(5)	119.7(3)
N(4)-C(4)-Yb(1)	164.9(2)	N(1)-C(4)-Yb(1)	43.1(1)
C(5)-C(4)-Yb(1)	77.1(2)	N(3)-C(5)-N(2)	124.6(2)
N(3)-C(5)-C(4)	115.5(3)	N(2)-C(5)-C(4)	119.9(2)
N(3)-C(5)-Yb(1)	165.1(2)	N(2)-C(5)-Yb(1)	43.0(1)
C(4)-C(5)-Yb(1)	77.3(2)	N(2)-C(6)-C(7)	122.5(3)
N(2)-C(6)-H(6)	118.8	C(7)-C(6)-H(6)	118.8
C(8)-C(7)-C(6)	117.3(3)	C(8)-C(7)-H(7)	121.3
C(6)-C(7)-H(7)	121.3	N(3)-C(8)-C(7)	123.5(3)
N(3)-C(8)-H(8)	118.3	C(7)-C(8)-H(8)	118.3
Ni(1)-C(9)-H(9A)	109.5	Ni(1)-C(9)-H(9B)	109.5
H(9A)-C(9)-H(9B)	109.5	Ni(1)-C(9)-H(9C)	109.5
H(9A)-C(9)-H(9C)	109.5	H(9B)-C(9)-H(9C)	109.5
Ni(1)-C(10)-H(10A)	109.5	Ni(1)-C(10)-H(10B)	109.5
H(10A)-C(10)-H(10B)	109.5	Ni(1)-C(10)-H(10C)	109.5
H(10A)-C(10)-H(10C)	109.5	H(10B)-C(10)-H(10C)	109.5
C(15)-C(11)-C(12)	108.2(3)	C(15)-C(11)-C(16)	125.2(3)
C(12)-C(11)-C(16)	126.4(3)	C(15)-C(11)-Yb(1)	74.5(2)
C(12)-C(11)-Yb(1)	74.6(2)	C(16)-C(11)-Yb(1)	120.6(2)
C(11)-C(12)-C(13)	107.5(3)	C(11)-C(12)-C(17)	125.4(3)
C(13)-C(12)-C(17)	126.8(3)	C(11)-C(12)-Yb(1)	73.7(2)
C(13)-C(12)-Yb(1)	74.2(2)	C(17)-C(12)-Yb(1)	122.8(2)
C(14)-C(13)-C(12)	108.5(3)	C(14)-C(13)-C(18)	126.0(3)
C(12)-C(13)-C(18)	125.1(3)	C(14)-C(13)-Yb(1)	74.4(2)
C(12)-C(13)-Yb(1)	74.1(2)	C(18)-C(13)-Yb(1)	123.2(2)
C(13)-C(14)-C(15)	107.6(3)	C(13)-C(14)-C(19)	123.3(3)
C(15)-C(14)-C(19)	128.4(3)	C(13)-C(14)-Yb(1)	74.3(2)
C(15)-C(14)-Yb(1)	73.8(2)	C(19)-C(14)-Yb(1)	125.4(2)
C(11)-C(15)-C(14)	108.2(3)	C(11)-C(15)-C(20)	124.4(3)
C(14)-C(15)-C(20)	126.7(3)	C(11)-C(15)-Yb(1)	74.1(2)
C(14)-C(15)-Yb(1)	74.4(2)	C(20)-C(15)-Yb(1)	125.2(2)

C(11)-C(16)-H(16A)	109.5	C(11)-C(16)-H(16B)	109.5
H(16A)-C(16)-H(16B)	109.5	C(11)-C(16)-H(16C)	109.5
H(16A)-C(16)-H(16C)	109.5	H(16B)-C(16)-H(16C)	109.5
C(12)-C(17)-H(17A)	109.5	C(12)-C(17)-H(17B)	109.5
H(17A)-C(17)-H(17B)	109.5	C(12)-C(17)-H(17C)	109.5
H(17A)-C(17)-H(17C)	109.5	H(17B)-C(17)-H(17C)	109.5
C(13)-C(18)-H(18A)	109.5	C(13)-C(18)-H(18B)	109.5
H(18A)-C(18)-H(18B)	109.5	C(13)-C(18)-H(18C)	109.5
H(18A)-C(18)-H(18C)	109.5	H(18B)-C(18)-H(18C)	109.5
C(14)-C(19)-H(19A)	109.5	C(14)-C(19)-H(19B)	109.5
H(19A)-C(19)-H(19B)	109.5	C(14)-C(19)-H(19C)	109.5
H(19A)-C(19)-H(19C)	109.5	H(19B)-C(19)-H(19C)	109.5
C(15)-C(20)-H(20A)	109.5	C(15)-C(20)-H(20B)	109.5
H(20A)-C(20)-H(20B)	109.5	C(15)-C(20)-H(20C)	109.5
H(20A)-C(20)-H(20C)	109.5	H(20B)-C(20)-H(20C)	109.5
C(25)-C(21)-C(22)	108.3(3)	C(25)-C(21)-C(26)	126.4(3)
C(22)-C(21)-C(26)	125.0(3)	C(25)-C(21)-Yb(1)	75.6(2)
C(22)-C(21)-Yb(1)	74.8(2)	C(26)-C(21)-Yb(1)	120.9(2)
C(21)-C(22)-C(23)	108.0(3)	C(21)-C(22)-C(27)	124.6(3)
C(23)-C(22)-C(27)	126.4(3)	C(21)-C(22)-Yb(1)	73.6(2)
C(23)-C(22)-Yb(1)	75.0(2)	C(27)-C(22)-Yb(1)	125.8(2)
C(24)-C(23)-C(22)	107.7(3)	C(24)-C(23)-C(28)	125.3(3)
C(22)-C(23)-C(28)	126.7(3)	C(24)-C(23)-Yb(1)	74.7(2)
C(22)-C(23)-Yb(1)	73.4(2)	C(28)-C(23)-Yb(1)	122.8(2)
C(23)-C(24)-C(25)	108.2(3)	C(23)-C(24)-C(29)	126.2(3)
C(25)-C(24)-C(29)	125.1(3)	C(23)-C(24)-Yb(1)	74.2(2)
C(25)-C(24)-Yb(1)	73.9(2)	C(29)-C(24)-Yb(1)	124.0(2)
C(21)-C(25)-C(24)	107.7(3)	C(21)-C(25)-C(30)	126.9(3)
C(24)-C(25)-C(30)	125.1(3)	C(21)-C(25)-Yb(1)	73.0(2)
C(24)-C(25)-Yb(1)	74.6(2)	C(30)-C(25)-Yb(1)	122.9(2)
C(21)-C(26)-H(26A)	109.5	C(21)-C(26)-H(26B)	109.5
H(26A)-C(26)-H(26B)	109.5	C(21)-C(26)-H(26C)	109.5
H(26A)-C(26)-H(26C)	109.5	H(26B)-C(26)-H(26C)	109.5
C(22)-C(27)-H(27A)	109.5	C(22)-C(27)-H(27B)	109.5
H(27A)-C(27)-H(27B)	109.5	C(22)-C(27)-H(27C)	109.5
H(27A)-C(27)-H(27C)	109.5	H(27B)-C(27)-H(27C)	109.5
C(23)-C(28)-H(28A)	109.5	C(23)-C(28)-H(28B)	109.5
H(28A)-C(28)-H(28B)	109.5	C(23)-C(28)-H(28C)	109.5
H(28A)-C(28)-H(28C)	109.5	H(28B)-C(28)-H(28C)	109.5
C(24)-C(29)-H(29A)	109.5	C(24)-C(29)-H(29B)	109.5
H(29A)-C(29)-H(29B)	109.5	C(24)-C(29)-H(29C)	109.5
H(29A)-C(29)-H(29C)	109.5	H(29B)-C(29)-H(29C)	109.5
C(25)-C(30)-H(30A)	109.5	C(25)-C(30)-H(30B)	109.5
H(30A)-C(30)-H(30B)	109.5	C(25)-C(30)-H(30C)	109.5
H(30A)-C(30)-H(30C)	109.5	H(30B)-C(30)-H(30C)	109.5
C(32)-C(31)-C(36)	120.0	C(32)-C(31)-H(31)	120.0
C(36)-C(31)-H(31)	120.0	C(31)-C(32)-C(33)	120.0
C(31)-C(32)-H(32)	120.0	C(33)-C(32)-H(32)	120.0
C(34)-C(33)-C(32)	120.0	C(34)-C(33)-H(33)	120.0
C(32)-C(33)-H(33)	120.0	C(35)-C(34)-C(33)	120.0
C(35)-C(34)-H(34)	120.0	C(33)-C(34)-H(34)	120.0
C(36)-C(35)-C(34)	120.0	C(36)-C(35)-H(35)	120.0
C(34)-C(35)-H(35)	120.0	C(35)-C(36)-C(31)	120.0
C(35)-C(36)-C(37)	121.0(5)	C(31)-C(36)-C(37)	120.0
C(36)-C(37)-H(37A)	109.5	C(36)-C(37)-H(37B)	119.0(5)
H(37A)-C(37)-H(37B)	109.5	C(36)-C(37)-H(37C)	109.5
H(37A)-C(37)-H(37C)	109.5	H(37B)-C(37)-H(37C)	109.5
C(44)-C(38)-C(39)	123.3(8)	C(44)-C(38)-C(43)	109.5

C(39)-C(38)-C(43)	120.0	C(38)-C(39)-C(40)	116.6(8)
C(38)-C(39)-H(39)	120.0	C(40)-C(39)-H(39)	120.0
C(41)-C(40)-C(39)	120.0	C(41)-C(40)-H(40)	120.0
C(39)-C(40)-H(40)	120.0	C(42)-C(41)-C(40)	120.0
C(42)-C(41)-H(41)	120.0	C(40)-C(41)-H(41)	120.0
C(43)-C(42)-C(41)	120.0	C(43)-C(42)-H(42)	120.0
C(41)-C(42)-H(42)	120.0	C(42)-C(43)-C(38)	120.0
C(42)-C(43)-H(43)	120.0	C(38)-C(43)-H(43)	120.0
C(38)-C(44)-H(44A)	109.5	C(38)-C(44)-H(44B)	109.5
H(44A)-C(44)-H(44B)	109.5	C(38)-C(44)-H(44C)	109.5
H(44A)-C(44)-H(44C)	109.5	H(44B)-C(44)-H(44C)	109.5

V. DFT Calculation data

Table S7. Average main distances (Å) and angles (°) for (bipym)$NiMe_2$ (**1**) DFT optimized geometry with three different functional (PBE, PBE0 and TPSSh) vs XRD crystal structure.

Atoms	PBE	PBE0	TPSSH	XRD
Ni-C	1.916	1.899	1.911	1.930(3)
Ni-N	1.951	1.967	1.953	1.959(2)
$C\text{-}C_{bipym}$	1.471	1.472	1.469	1.482(5) (avg)
Me-Ni-Me^N-Ni-N	9.41	16.90	13.11	5.34±0.24 (avg)
$N\text{-}C\text{-}C\text{^}C\text{-}C\text{-}N_{bipym}$	4.65	12.80	7.64	2.63±1.04 (avg)

Table S8. Cartesian coordinates for the PBE optimized geometry.

Ni	1.02122660532342	3.14409386460801	2.98955432851006
N	3.91360444234729	4.93868469680804	5.32137831697165
N	3.15486436792260	6.66722934684548	3.21831859757962
N	1.56176679775467	4.97442958846558	2.58837429646529
N	2.38962225339262	3.33566713372091	4.36642759969447
C	4.24033898716970	4.04773961720592	6.26366464340771
H	4.98635783793639	4.36428405231481	6.99768060213871
C	3.65817056844596	2.77731679180689	6.31420239951038
H	3.92285178249195	2.05589718569941	7.08704014023842
C	2.72935094484467	2.45760899158293	5.33507856458957
H	2.23548155386991	1.48667871576435	5.29492938935872
C	3.00803549169623	4.55670098436277	4.42293231353177
C	2.57243883180281	5.47874198663747	3.36302047684962
C	2.71837091578446	7.43387918165066	2.21363972299956
H	3.19828821478854	8.40933797760293	2.09967315768061
C	1.70361040797094	7.01292402540281	1.34882886595659
H	1.35468563617542	7.63796199984575	0.52717989197954
C	1.14453435266755	5.76481774611270	1.57551309124557
H	0.34351324663976	5.36335004401491	0.95438667140380
C	0.70410436388021	1.28121597496656	3.30689149168597
H	0.14641613697265	1.19840544311913	4.25767278362353
H	1.68857053585856	0.79228394014465	3.41559034905400
H	0.13895701126033	0.74582915917873	2.53447105491620
C	-0.44491390658014	3.12339516530584	1.75634308650510
H	-1.14860290574047	2.28621375428331	1.84072090323621
H	-0.02013525688884	3.11394692159549	0.73612381698628
H	-1.00372921778722	4.06404571095393	1.90861344388103

Table S9. Cartesian coordinates for the PBE0 optimized geometry.

Ni	1.02443198505548	3.15381408738126	2.98059621977688
N	3.73407525725889	5.00466505653483	5.43642947631058
N	3.18575891561773	6.63363577128596	3.19729907056606
N	1.55302893115246	5.00632272424439	2.62031668191389
N	2.45510586297498	3.33513723346412	4.32643779433853
C	4.06350802992681	4.11545020947509	6.36353847881887
H	4.69037321483515	4.47141138993181	7.17537347015335
C	3.63115049964051	2.79903385436962	6.30812635732142
H	3.91447565701858	2.07428781708130	7.05975021476159
C	2.81024627462597	2.44674676479416	5.25548582068772
H	2.41085318536828	1.44713263577522	5.13513904654961
C	2.95819026798853	4.57663390851985	4.45976699545082
C	2.55293522753717	5.48968112970821	3.37824140127253
C	2.80725810939503	7.35861677232767	2.15364199650896
H	3.33727808606635	8.29256317038145	1.99411869474128
C	1.79410256272804	6.95090265262687	1.29916782856216
H	1.49344416245264	7.54650925452393	0.44785272305163
C	1.17950335068019	5.74683079492121	1.57701659717732
H	0.37721104287126	5.34426999229061	0.97182426951632
C	0.83053440953936	1.27444589676548	3.13924331453817
H	0.31931136020791	1.01679031668262	4.07758537248421
H	1.83248223232069	0.82065452606085	3.15418392128216
H	0.27112600088362	0.80468916646153	2.32902257643419
C	-0.57913007981063	3.18784299600823	1.96021117695315
H	-1.32674752923950	2.47785926291177	2.31896414441347
H	-0.37627170464102	2.94575176395743	0.90816459300438
H	-1.02645531245451	4.19100085151452	2.01075176341074

Table S10. Cartesian coordinates for the TPSSh optimized geometry.

Ni	1.01605874053002	3.15477826488399	2.99669812615423
N	3.86704470965689	4.96096384452984	5.35497278496369
N	3.18150292980035	6.64880191416881	3.19967284837824
N	1.55160589242891	4.99333273570274	2.61596439052871
N	2.40366529576587	3.34566461877816	4.35973718090016
C	4.18560017616430	4.07078468599449	6.29410080051198
H	4.89601844054177	4.39665081628832	7.04686425276797
C	3.63532707385691	2.79221511867931	6.31671959706934
H	3.89780153567076	2.07335897009374	7.08190938885243
C	2.73770147724413	2.46427241142651	5.31772336778343
H	2.26524921009032	1.49320241870463	5.25392536773564
C	2.99522207905912	4.56722353687998	4.43585137475829
C	2.57047744793116	5.48372486845664	3.36989522093211
C	2.76167266368714	7.39843727167811	2.18167857584365
H	3.26654877516045	8.34855344900602	2.04168379070249
C	1.73499144951792	6.98764567244854	1.33580978436789
H	1.40251853856117	7.59990097728904	0.50789987016247
C	1.14669310547595	5.76353058017209	1.59210476045569
H	0.33963119896529	5.36641163604271	0.99090609782762
C	0.77469305113230	1.27388418459435	3.22721279053762
H	0.17945683952117	1.09200622496433	4.13321269253687
H	1.76443554620180	0.81246360283823	3.35449762963640

H	0.27557743695802	0.77326910405294	2.39630000065619
C	-0.50818958816696	3.14779017566527	1.84214773622614
H	-1.23430033281012	2.35989583898908	2.04821832267607
H	-0.16866493171474	3.02827840533922	0.80324943066894
H	-1.02055876122990	4.11563867233286	1.93929381636574

Table S11. Single point energy difference (kcal/mol) vs the PBE_{gas} calculated one. All these single point energies were calculated starting from the PBE optimized geometry. Two environments are studied: in gas phase and with the presence of a toluene *continuum* (CPCM method).

	PBE_{gas}	$PBE0_{gas}$	$TPSSh_{gas}$	PBE_{Tol}	$PBE0_{Tol}$	$TPSSh_{Tol}$
ΔSPE(PBE_{gas}) – SPE	0	18.095	-636.954	-14.585	4.850	-649.198

Table S12. MO energy gaps (cm^{-1}) calculated with three different functionals (PBE, PBE0 and TPSSh) starting from the PBE optimized geometry. Two environments are studied: in gas phase and with the presence of a toluene *continuum* (CPCM method).

MO	PBE_{gas}	$PBE0_{gas}$	$TPSSh_{gas}$	PBE_{Tol}	$PBE0_{Tol}$	$TPSSh_{Tol}$
HOMO => LUMO	7260.22	22324.96	13758.86	8072.28	23975.41	14856.24
LUMO => LUMO +1	4299.51	5366.15	4903.06	4378.52	5469.31	5006.216

PBE_{gas}

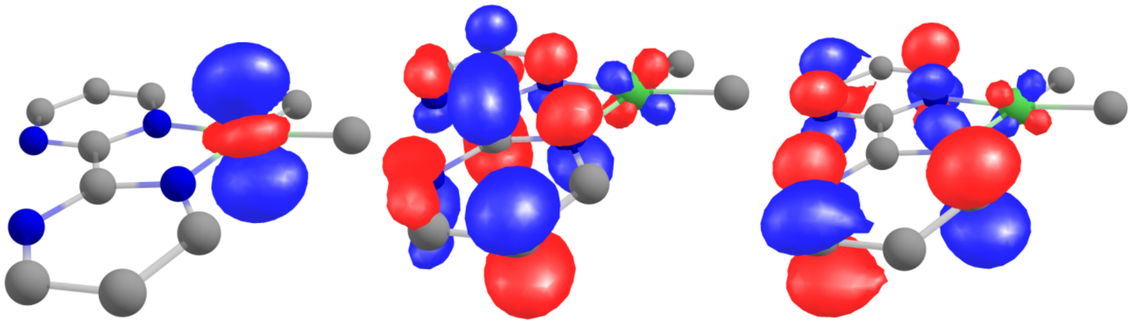

$PBE0_{gas}$

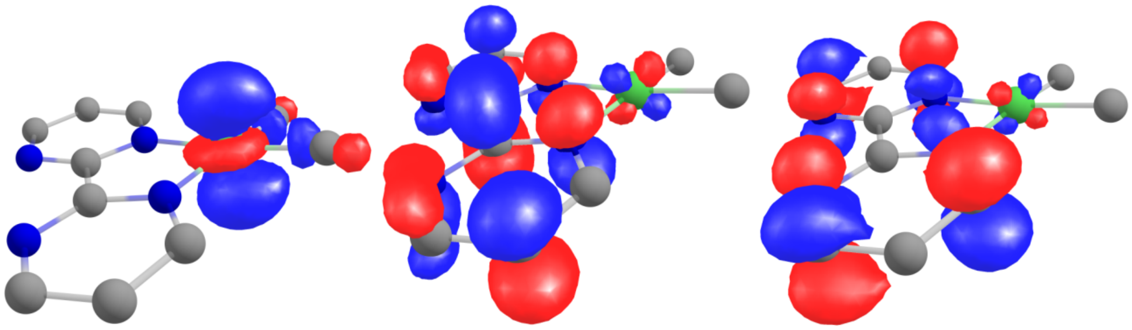

$TPSSh_{gas}$

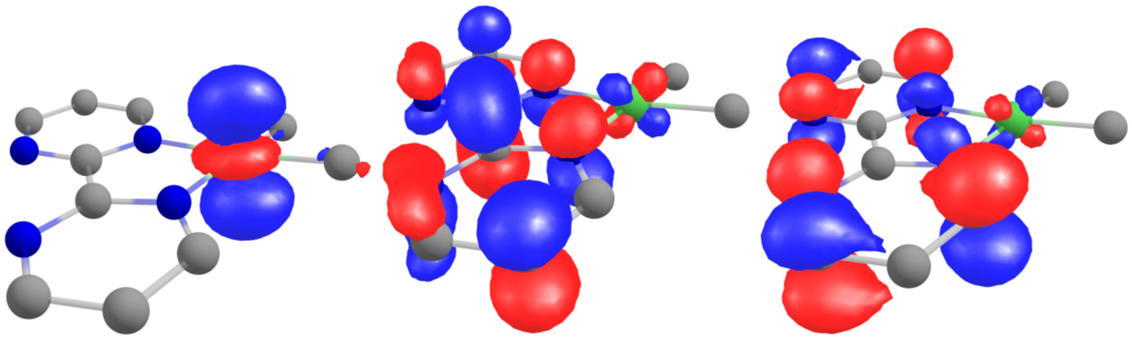

Figure S24. Kohn-Sham orbitals obtained with three different functionals (PBE, PBE0 and TPSSh) starting from the PBE optimized geometry in gas phase.

PBE$_{Tol}$

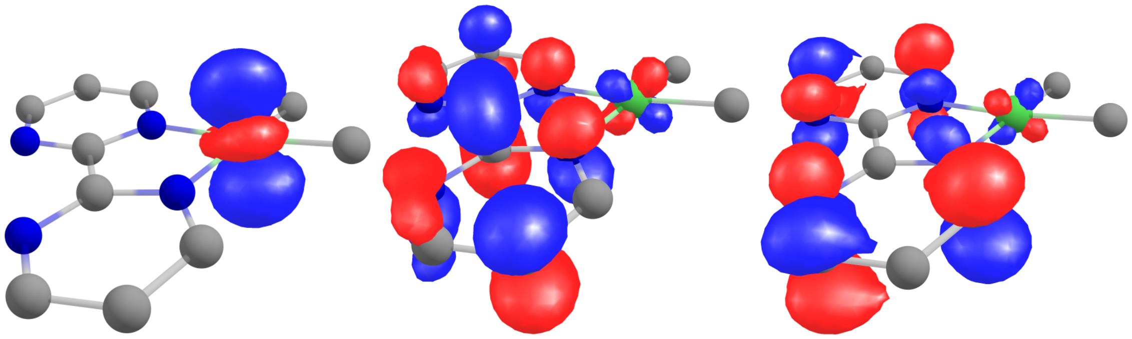

PBE0$_{Tol}$

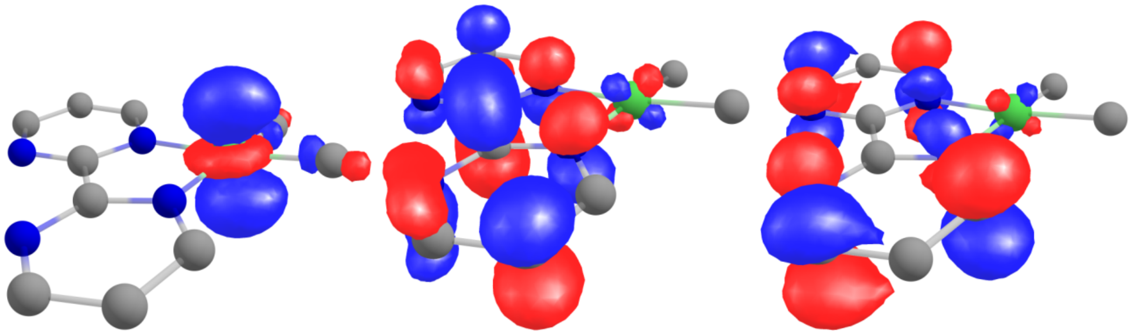

TPSSh$_{Tol}$

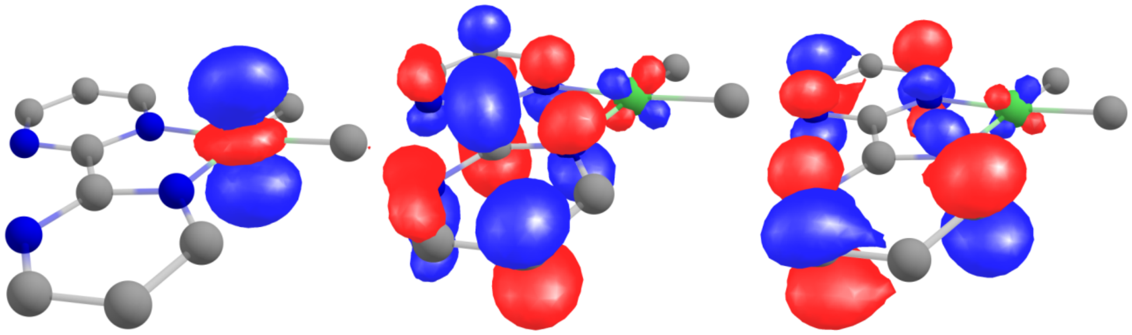

Figure S25. Kohn-Sham orbitals obtained with three different functionals (PBE, PBE0 and TPSSh) starting from the PBE optimized geometry and using a toluene *continuum* (CPCM method).
